# D-mannose is a rapid inducer of ACSS2 to trigger rapid and long-lasting antidepressant responses through augmenting BDNF and TPH2 levels

**DOI:** 10.1038/s41398-023-02636-7

**Published:** 2023-11-01

**Authors:** Nuo Chen, Ming Zhao, Yaxin Guo, Nan Wu, Baihui Cao, Bing Zhan, Tian Zhou, Yubin Li, Faliang Zhu, WanJun Chen, Yan Li, Lining Zhang

**Affiliations:** 1 Department of Immunology, School of Basic Medical Science, Cheeloo College of Medicine, Shandong University, Jinan, China; 2 Department of Pathogen Biology, School of Basic Medical Science, Cheeloo College of Medicine, Shandong University, Jinan, China; 3 Mucosal Immunology Section, NIDCR, US National Institutes of Health, Bethesda, MD USA

**Keywords:** Depression

## Abstract

The potentiation of synaptic plasticity and serotonin generation by brain-derived neurotrophic factor (BDNF) and tryptophan hydroxylase 2 (TPH2) is well characterized to facilitate rapid and long-lasting antidepressant actions. Therefore, the identification of the key protein that simultaneously controls both BDNF and TPH2 is important for the treatment of depression. We show here that a lack of acetyl-CoA synthetase short-chain family member 2 (ACSS2) causes impairments in BDNF-dependent synaptic plasticity and tryptophan hydroxylase 2 (TPH2)-mediated serotonin generation, thereby contributing to spontaneous and chronic restraint stress (CRS)-induced depressive-like behavior in mice. Conversely, D-mannose is identified as a rapid ACSS2 inducer and thus mediates rapid and long-lasting antidepressant-like effects. Mechanistically, acute and chronic D-mannose administration inhibits the phosphorylation of EF2 to increase BDNF levels and reverse the reduction of *TPH2* histone acetylation and transcription. We reveal that ACSS2 promotes *TPH2* histone acetylation and transcription with the requirement of AMPK activation. To elevate nuclear ACSS2 levels, D-mannose can rapidly and persistently activate AMPK via Ca^2+^-CAMKK2 and the lysosomal AXIN-LKB1 pathway to facilitate its fast-acting and persistent antidepressant responses. Taken together, the results presented here reveal that ACSS2 functions as a novel target to link rapid and persistent antidepressant actions and further suggest that D-mannose is a potential therapeutic agent to resist depression through its augmentation of the ACSS2 dependent BDNF and TPH2 pathways.

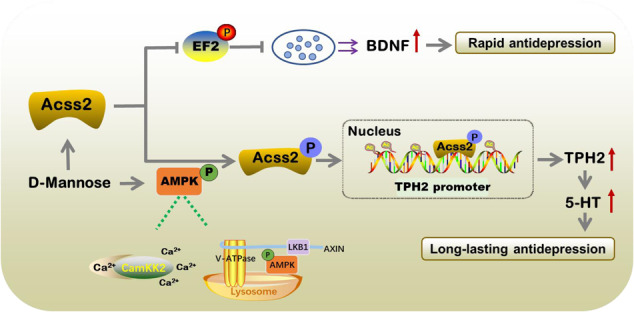

## Introduction

The metabolic enzymes that generate acetyl-CoA can control lipid synthesis and directly sense intracellular acetyl-CoA alterations to regulate chromatin remodeling and gene expression through histone acetylation [1, 2]. The phosphorylation of acetyl-CoA synthetase 2 (ACSS2) on serine 659 via AMPK allows the enzyme to enter the nucleus and locally produce acetyl-CoA to favor histone acetylation of lysosomal and autophagy genes [3]. Although ACSS2 is highly expressed in the hippocampus and serves as a key histone acetylation modulator in regulating hippocampal neuron memory and alcohol metabolism [4, 5], the role of ACSS2 in emotion control has yet to be determined.

Major depressive disorder is a leading cause of disability worldwide with diverse symptoms that severely influence quality of life [6, 7]. During depression pathogenesis, synaptic transmission is often impaired; thus, brain-derived neurotrophic factor (BDNF), which plays an important role in adult neurogenesis, neuronal maturation and synaptic plasticity, has been extensively recognized as a target to resist depression [8–12]. In particular, BDNF is required for the ketamine-mediated rapid antidepressant response to promote synaptic formation [13–15]. During this process, ketamine reduces the level of eEF2 phosphorylation to permit BDNF protein synthesis. However, the potential mediators of BDNF-mediated antidepressant effects remain unclear [16]. The activation of tropomyosin receptor kinase B by BDNF leads to activations of three intracellular signaling pathways including the phospholipase Cγ-IP3/Ca^2+^/protein kinase C, ERK-mitogen-activated protein kinase (MAPK) and phosphatidylinositol-3-kinase (PI3K)-AKT-mTOR pathways [17]. The findings of some reports support that the antidepressant action of BDNF might depend on ERK pathway via MAPK phosphorylation [18–20]. However, there are several findings to indicate that the AKT-mTOR pathway may also be involved in the BDNF-mediated antidepressant response [19, 21]. Nonetheless, there is no doubt that rapid elevation of BDNF is a crucial contributor to fast-acting and long-term antidepressant responses [22].

Due to its long-lasting antidepressant effects, tryptophan hydroxylase 2 (TPH2), which is the rate-limiting enzyme for brain serotonin (5-hydroxytryptamine, 5-HT) biosynthesis, has been shown to be a promising target for the therapeutic treatment of neuropsychiatric disorders including depression and anxiety due to the close relationship between 5-HT system dysfunction and depression [23–26]. Multiple environmental factors such as stressful events (internal or external), endogenous hormones or growth factors, diet and exogenous drugs or chemicals influence TPH2 expression [27]. Neuron-restrictive silencer element, neuron-restrictive silencer factor, Sin3A and class I histone deacetylase (HDAC) constitute the corepressor complex that suppresses *TPH2* transcription [28]. However, the precise mechanism of *TPH2* transcriptional activation remains largely unknown.

Here, we show that ACSS2 controls fast-acting and long-lasting antidepressant responses by augmenting BDNF and TPH2 levels. To support the potential of ACSS2 as a therapeutic target for depression, D-mannose was identified as a rapid ACSS2 inducer that increases synaptic plasticity and serotonin generation, thereby mediating rapid and persistent antidepressive-like behaviors in mice. In response to acute and chronic D-mannose administration, eEF2 phosphorylation was suppressed to facilitate rapid BDNF elevation while TPH2 transcription was activated to promote serotonin generation in an ACSS2-dependent manner upon AMPK activation. Moreover, D-mannose can activate AMPK through Ca^2+^-CAMKK2- and lysosomal AXIN-LKB1-dependent pathways to elevate nuclear ACSS2 levels. The downregulation of hippocampal ACSS2 abolished the antidepressant effect of D-mannose. We thus reveal a previously unrecognized ACSS2 function as a novel rapid and long-lasting antidepressant target by upregulating BDNF and TPH2. This novel information has clinical implications for the prevention and treatment of human depression and other metabolic diseases by targeting ACSS2.

## Results

### Lack of ACSS2 causes defects in synaptic formation and reduces levels of BDNF and TPH2 to induce depressive symptoms

**Fig. 1:**
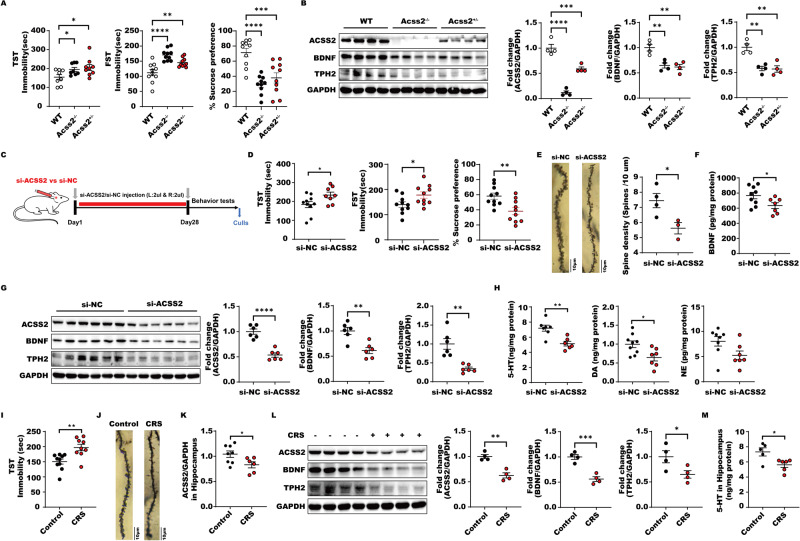
Lack of ACSS2 causes defects of synaptic formation and reduces levels of BDNF and TPH2 to induce depressive symptoms. **A** Immobility time in the tail suspension test, forced swimming test, and sucrose consumption in the sucrose preference test from male mice in individual animals from wild type, *Acss2*^*−/−*^ and *Acss2*^*+/−*^ groups (*n* = 8–10). **B** Representative immunoblots and quantification of hippocampal ACSS2, BDNF, TPH2 protein levels normalized to loading controls (*n* = 4) from wild type, *Acss2*^*−/−*^ and Acss2^+/−^ male mice. **C** C57BL/6 J male mice were injected with lentivirus of ACSS2 or control group with NC (2μl was injected into the left and right hippocampus respectively; *n* =8–10 male mice per group) in stereotactic location in the hippocampus. TST, FST, and SPT behavioral tests were performed 4 weeks after the injection of lentivirus. **D** Immobility time in the tail suspension test, forced swimming test, and sucrose consumption in the sucrose preference test from male mice in individual animals from si-NC and si-ACSS2 groups (*n* =8–10). **E** Representative photomicrographs of dendritic spines from DG granular cells, scale bar, 10 μm. And Spine density in dendrites of DG granular cells, (si-NC *n* = 4 and siACSS2 *n* = 3 per group). Values are indicated as mean ± SD one-way ANOVA and multiple comparison test, *P ≤ 0.05 **P ≤ 0.01. All the experiments were repeated at least three times independently. **F** Analysis of BDNF content of HIP by ELISA in male mice from si-NC and si-ACSS2 groups (*n* = 7–9). **G** Representative immunoblots and quantification of hippocampal ACSS2, BDNF, TPH2 protein levels normalized to loading controls (*n* = 6) from si-ACSS2 male mice. **H** Analysis of 5-HT, DA and NA/NE content of HIP by ELISA in male mice from si-NC and si-ACSS2 groups (*n* = 6–9). **I** Immobility time in the tail suspension test from male mice in individual animals from control and CRS groups (*n* =9–10). **J** Representative photomicrographs of dendritic spines from DG granular cells, scale bar, 10 μm. **K** RT-PCR analysis of *ACSS2* expression levels in Hip of male mice (*n* =6–8) exposure to Control and CRS. Data were normalized with GAPDH mRNA levels and presented as fold changes compared with control group. Scale bars represent mean values and error bars represent SEM. **L** Representative immunoblots and quantification of hippocampal ACSS2, BDNF, TPH2 protein levels normalized to loading controls (*n* = 4) from Control and CRS male mice. **M** Analysis of 5-HT content of HIP by ELISA in male mice from Control and CRS groups (*n* =5–6).

To investigate the function of ACSS2 in depression pathogenesis, we first evaluated the behavioral phenotypes of *Acss2*^*+/−*^ and *Acss2*^*−/−*^ mice, and we observed that both displayed anxiety- and depressive-like behaviors in the open field test (OFT), elevated plus-maze test (EPM), tail suspension test (TST), forced swim test (FST) and sucrose preference test (SPT) compared to their wild type (WT) littermates, with subtle changes in body weight, and food and water intake, suggesting that reduced ACSS2 levels might cause spontaneous depression-like behavior in mice (Fig. 1A, B, S1A–E). Importantly, we observed that the hippocampal levels of BDNF and TPH2 were significantly reduced in *Acss2*^*+/−*^ and *Acss2*^*−/−*^ mice compared with WT mice, indicating that ACSS2 deficiency might cause both synapse impairment and serotonin transmitter dysfunction.

Consistent with these findings, specific ACSS2 downregulation in the hippocampus through stereotactic injection of a lentivirus carrying small interfering RNA targeting *Acss2* also caused anxiety- and depressive-like behaviors in the OFT, EPM, TST, FST, and SPT (Fig. 1C, D, S1F, G). The spine density and number of spines in DG granule neurons were reduced in response to ACSS2 downregulation (Fig. 1E). Correspondingly, BDNF levels were significantly decreased in response to silencing ACSS2 (Fig. 1F, G). Similar to ACSS2 knockout mice, the hippocampal levels of TPH2 and 5-HT were reduced in response to ACSS2 downregulation (Fig. 1G, H). Of note, other brain neurotransmitters, such as dopamine (DA) and norepinephrine (NE), in the hippocampus also tended to decline. The above data suggest that, ACSS2 insufficiency or deficiency leads to synaptic formation defects and serotonin transmission dysfunction.

In mice, mRNA and protein levels of ACSS2 in the hippocampus, along with the typical behavioral phenotypes of depression, were reduced in response to chronic restraint stress (CRS)-induced depressive-like behaviors, as assessed by the TST (Fig. 1I, K, L, S1H). In line with the above observations, the spine density and number in DG granule neurons as well as hippocampal BDNF levels were significantly decreased in mice with CRS-induced depressive-like behaviors (Fig. 1J, L). The hippocampal levels of TPH2 and 5-HT were also reduced in CRS mice (Fig. 1L, M). In summary, these data support that a lack of ACSS2 contributes to defects in synaptic formation, and BDNF and serotonin generation via TPH2, thereby contributing to spontaneous and CRS-induced depressive-like behaviors in mice.

### Acute D-mannose administration possesses fast-acting antidepressant activity via ACSS2

**Fig. 2:**
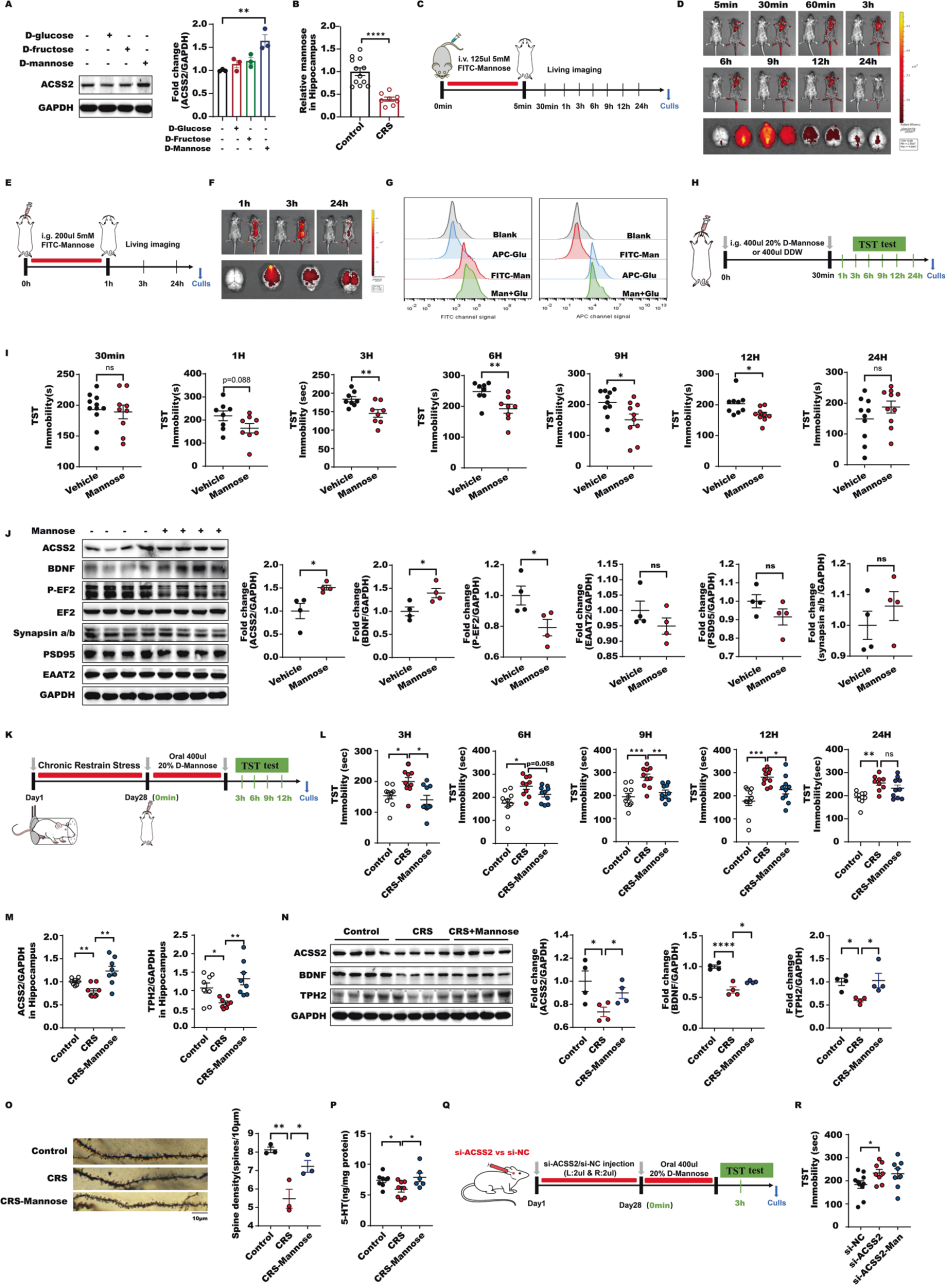
Acute D-mannose administration possesses fast-acting antidepressant activity via ACSS2. **A** Immunoblot analyses of SH-SY5Y cells expressing ACSS2 with 1 mM D-glucose, 1 mM D-fructose or 1 mM D-Mannose for 24 h with the indicated antibodies were performed. **B** Analysis of D-Mannose in hippocampus (Hip) in response to chronic restrain stress (CRS) in male mice (*n* = 8–12). The D-Mannose levels were normalized to those in Control male mice. **C** 125ul 5 mM FITC-D-Mannose was injected into male mice via tail vein, the intensity and distribution of red fluorescence were detected by the small animal three-dimensional live imaging system at 5 min, 30 min, 60 min, 3 h, 6 h, 9 h, 12 h and 24 h after injection. **D** 125ul 5 mM FITC-D-Mannose was injected into male mice via tail vein, the intensity and distribution of red fluorescence were detected by the small animal three-dimensional live imaging system at 5 min, 30 min, 60 min, 3 h, 6 h, 9 h, 12 h and 24 h after injection. **E** 200ul 5 mM FITC-D-Mannose was injected into male mice via gavage, the intensity and distribution of red fluorescence were detected by the small animal three-dimensional live imaging system at 1 h, three h and 24 h after injection. **F** 200 μl 5 mM FITC-D-Mannose was injected into male mice via gavage, the intensity and distribution of red fluorescence were detected by the small animal three-dimensional live imaging system at 1 h, 3 h and 24 h after injection. **G** SH-SY5Y cells were incubated with 20 μM FITC labeled D-Mannose and CY5 tagged glucose alone or together followed by flow cytometry analysis. **H** 400 μl 20% D-Mannose or 400 μl water were injected into male mice via gavage and TST behavior test was detected at 30 min, 1 h, 3 h, 6 h, 9 h, 12 h and 24 h after injection. **I** Immobility time in the tail suspension test from male mice in individual animals from wild type and D-Mannose supplementation groups (*n* ≥ 8) at 30 min, 1 h, 3 h, 6 h, 9 h, 12 h and 24 h after intragastric administration of 400 μl water or 20% D-Mannose. **J** Representative immunoblots and quantification of hippocampal ACSS2, BDNF, P-EF2, EF2, Synapsin a/b, PSD95, and EAAT2 protein levels normalized to loading controls (*n* = 4) from D-Mannose supplementation male mice. **K** C57BL/6 J male mice suffered from chronic binding stress for 4 weeks, 400 μl 20% D-Mannose or 400 μl water were injected into male mice via gavage on Day29 and TST behavior test was detected at 3 h, 6 h, 9 h, 12 h and 24 h after injection. **L** Immobility time in the tail suspension test from male mice in individual animals from wild type, CRS and CRS-D-Mannose groups (*n* = 10) at 3 h, 6 h, 9 h,12 h and 24 h after intragastric administration of 400 μl water or 20% D-Mannose. **M** RT-PCR analysis of *ACSS2* and *TPH2* expression levels in Hip of male mice (*n* = 8) exposure to Control, CRS and CRS-D-Mannose. Data were normalized with GAPDH mRNA levels and presented as fold changes compared with control group. Scale bars represent mean values and error bars represent SEM. **N** Representative immunoblots and quantification of hippocampal ACSS2, BDNF, and TPH2 protein levels normalized to loading controls (*n* = 4) from male mice exposure to CRS and D-Mannose. **O** Representative photomicrographs of dendritic spines from DG granular cells, scale bar, 10 μm. And Spine density in dendrites of DG granular cells, (Control *n* = 3, CRS *n* = 3 and CRS-D-Mannose *n* = 3 per group). Values are indicated as mean ± SD one-way ANOVA and multiple comparison test, *P ≤ 0.05 **P ≤ 0.01. All the experiments were repeated at least three times independently. **P** Analysis of 5-HT content of HIP by ELISA in male mice from Control, CRS, and CRS-D-Mannose groups (*n* = 6–8). **Q** C57BL/6J male mice were injected with lentivirus of ACSS2 or control group with NC (2 μl was injected into the left and right hippocampus respectively; *n* = 8–10 mice per group) in stereotactic location in the hippocampus. 400 μl 20% D-Mannose or 400 μl water were injected into male mice via gavage on Day29 and TST behavior test was detected at 3 h after injection. **R** Immobility time in the tail suspension test from male mice in individual animals from si-NC, si-ACSS2, and si-ACSS2-D-Mannose groups (*n* = 8–10).

As we described above, ACSS2 insufficiency or deficiency can increase the risk of depression pathogenesis. To support it as an antidepressant effect target, we screened the ability of diverse hexoses, such as D-glucose, D-fructose and D-mannose, which are abundantly present in the ordinary diet of humans, to enhance ACSS2 expression in resisting depression. The results showed that D-mannose harbored the highest ability to increase ACSS2 expression compared with D-glucose and D-fructose (Fig. 2A). Furthermore, the D-mannose level in the hippocampus of mice with CRS-induced depressive-like behaviors was markedly reduced, suggesting that the decline in D-mannose may contribute to depression pathogenesis (Fig. 2B). Based on these findings, we focused on D-mannose and evaluated its antidepressant action. We found that D-mannose specifically entered the mouse brain within 5 min following a tail vein injection (TVI) and was persistently present until 24 h postinjection, indicating that D-mannose can quickly enter the brain but be metabolized at lower rates with a longer duration (Fig. 2C, D). In contrast, D-glucose seemed to be absorbed by all the tissues, whereas its metabolites also persisted for 24 h after TVI (Fig. S2A). These observations demonstrate that D-mannose, unlike D-glucose, may specifically and rapidly enter the brain and persists for a longer time. In line with these findings, we observed that D-mannose was able to reach the brain 1 h after a single oral gavage and was still present 24 h after injection (Fig. 2E, F). Moreover, D-mannose possessed the ability to enter human neuron-like cells (i.e., a human SH-SY5Y neuroblastoma cell line) without influencing cellular D-glucose uptake, suggesting that D-mannose can be utilized by neurons without impacting D-glucose transport, whereas they share the same sugar transporters (Fig. 2G). Next, we evaluated the acute effect of D-mannose on antidepressant activity in WT C57BL/6 mice. First, D-mannose did not have an obvious effect on hippocampal weight or peripheral tissue dysfunction, such as in heart, liver, spleen, lung and kidney tissue, in male or female mice (Fig. S2B–E). Interestingly, a single oral gavage of 400 μL of 20% D-mannose for 3 h started to significantly decrease the immobility of mice in the TST, and this antidepressant effect persisted for 12 h postinjection and disappeared at 24 h (Fig. 2H, I). eEF2 phosphorylation was significantly suppressed, and BDNF levels were markedly increased along with elevated ACSS2, whereas other factors associated with rapid antidepressant responses, such as the proteins PSD95, synapsin a/b and EAAT2 and glutamate content, were not changed (Fig. 2J, S2F). Moreover, the spine number in DG granule neurons displayed no alterations in response to a single D-mannose treatment (Fig. S2G). These observations support that D-mannose functions as a safe and fast-acting antidepressant agent. More importantly, the administration of a single dose of D-mannose markedly reduced the immobility of mice with CRS-induced depressive-like behaviors in the TST from 3 h posttreatment to 12 h and led to higher ACSS2, BDNF, and TPH2 levels, and these antidepressant effects disappeared until 24 h after treatment with acute D-mannose (Fig. 2K–N). These findings support that the behaviors of mice with depressive-like behaviors are rapidly and long-lasting improved by D-mannose, with the restoration of reduced BDNF and TPH2 levels. Accordingly, the decreases in spine number in DG granule neurons and 5-HT in the hippocampus were both reversed by D-mannose in mice with CRS-induced depressive-like behaviors (Fig. 2O, P). Conversely, the downregulation of ACSS2 in the hippocampus abolished D-mannose antidepressant activity, indicating that the D-mannose-mediated antidepressant effect is dependent on ACSS2 (Fig. 2Q, R). Collectively, these findings show that D-mannose rapidly enters the brain to induce ACSS2 expression, thereby augmenting BDNF and TPH2 levels to mediate fast-acting antidepressant action.

### Oral administration of D-mannose has long-lasting antidepressant actions by activating the ACSS2-TPH2 axis in mice with CRS-induced depressive-like behaviors

**Fig. 3:**
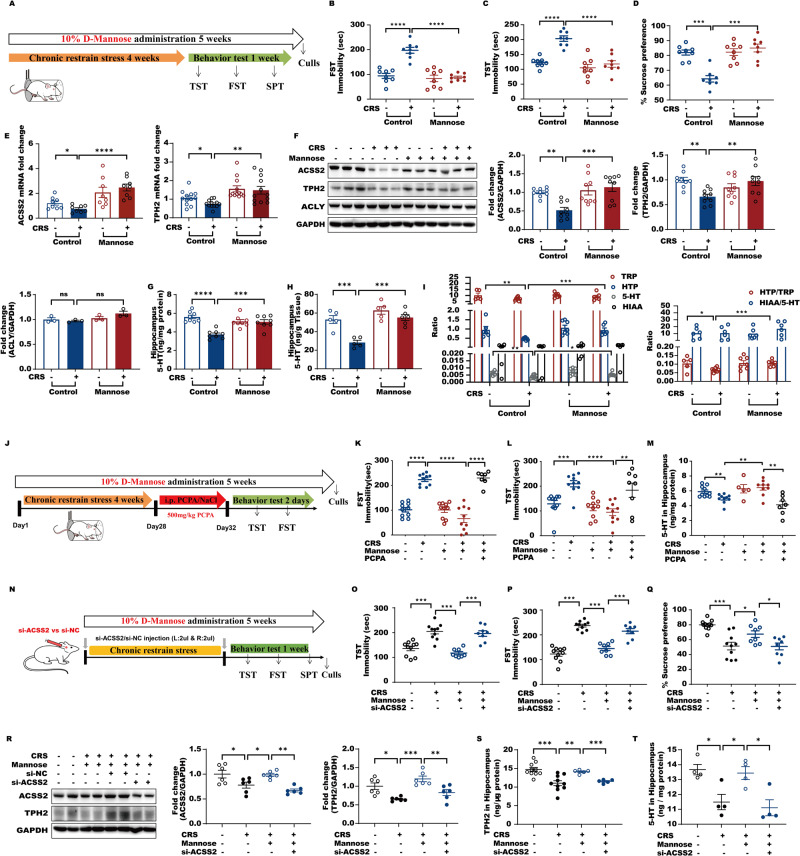
Oral administration of D-mannose has long-lasting antidepressant actions by activating the ACSS2-TPH2 axis in mice with CRS-induced depressive-like behaviors. **A** CRS-induced male mice with depressive-like behaviors for 4 weeks and male mice with or without 10% D-Mannose supplementation (*n* = 8) and behavioral testing started on Day29 and lasted for 1 week. **B** Immobility time in the forced swim test and tail suspension test **C** in individual animals (*n* = 8). **D** Sucrose preference in the presence of CRS and D-Mannose in male mice (*n* = 8). **E** RT-PCR analysis of *ACSS2* and *TPH2* expression levels in Hip of male mice (*n* = 8) exposure to CRS and D-Mannose. Data were normalized with GAPDH mRNA levels and presented as fold changes compared with control group. Scale bars represent mean values and error bars represent SEM. **F** Representative immunoblots and quantification of hippocampal ACSS2, TPH2, ACLY protein levels normalized to loading controls (*n* = 9) from male mice exposure to CRS and D-Mannose. **G** Analysis of 5-HT content of HIP by ELISA in male mice with CRS and D-Mannose supplementation (*n* = 8). **H** Measurement of serotonin (5-HT) in Hip in CRS-induced male mice with depressive-like behaviors with or without D-Mannose supplementation (*n* = 5–6) by high performance liquid chromatography (HPLC). **I** Analysis of TRP, 5-hydroxytryptophan (HTP), 5-HT and 5-hydroxindole acetic acid (HIAA) in hippocampus from Control, CRS-Control, D-Mannose and CRS-D-Mannose groups of male mice (*n* = 5–7). HTP/TRP and HIAA/5-HT ratios were shown to indicate TPH2 and MAO-A enzyme activity. **J** C57BL/6J male mice were divided into five groups: Control, CRS-Control, D-Mannose, CRS-D-Mannose, and CRS-D-Mannose with PCPA injection groups (*n* = 7–10). Male mice with 500 mg/kg PCPA or saline for four consecutive days and the behavioral experiments were performed at 2 h after the last injection. **K** Immobility time in the forced swimming test and tail suspension test (**L**) in Control, CRS-Control, D-Mannose, CRS-D-Mannose and CRS-D-Mannose with PCPA injection groups (*n* = 7–10). **M** Analysis of 5-HT content of HIP by ELISA in male mice from Control, CRS-Control, D-Mannose, CRS-D-Mannose and CRS-D-Mannose with PCPA injection groups (*n* = 5–10). **N** C57BL/6J male mice were divided into four groups: Control, CRS-Control, CRS-D-Mannose, CRS-D-Mannose-SiACSS2 groups (*n* = 8–10). And behavioral testing started on Day29 and lasted for 1 week. **O** Immobility time in the forced swimming test and tail suspension test (**P**) in individual animals (*n* = 8) from Control, CRS-Control, CRS-D-Mannose, CRS-D-Mannose-SiACSS2 groups (*n* = 8–10). **Q** Sucrose preference in the presence of CRS and D-Mannose with or without ACSS2 downregulation by small RNA interference in male mice (*n* = 8–10). **R** Representative immunoblots and quantification of hippocampal ACSS2 and TPH2 protein levels normalized to loading controls (*n* = 6) from male mice exposure to CRS and D-Mannose with or without ACSS2 downregulation. **S** The protein levels of TPH2 in Hip of male mice (*n* = 5–10) and 5-HT (**T**) in Hip of male mice (*n* = 4) from Control, CRS-Control, CRS-D-Mannose, CRS-D-Mannose-SiACSS2 groups by ELISA assay.

Since 5-HT is a well characterized neurotransmitter that mediates long-lasting antidepressant effect, we further assessed the effect of chronic D-mannose on persistently protecting mice with depressive-like behaviors. Considering that D-mannose can prevent glycolysis and facilitate ROS generation, we first examined hippocampal ATP and ROS changes and observed that they were not influenced by D-mannose administration (Fig. S3A, B). In addition, chronic administration of D-mannose did not have obvious effects on hippocampal weight or peripheral tissue dysfunction, such as in heart, liver, spleen, lung and kidney tissue, in male and female mice (Fig. S3C–I). These data supported that chronic D-mannose administration did not impact hippocampal energy or redox state. The behavioral tests showed that oral administration of supraphysiological amounts of D-mannose (10%) in water led to anti-anxiety and anti-depressive effects in male and female mice with CRS and chronic unpredictable mild stress male and female mice (Fig. 3A–D, S4A–E, S5A–E, S6A–E). Furthermore, chronic D-mannose administration reversed ACSS2 reductions in mice with depressive-like behaviors without influencing another acetyl-CoA synthetase, ACLY (Fig. 3E, F, S7A–C). These data suggest that D-mannose has general anti-depressive effects independent of sex. Then, we chose CRS male mice for further mechanistic investigation of the antidepressant effects of D-mannose. Notably, consistent with acute D-mannose administration, the reductions in TPH2 and 5-HT in the hippocampus of mice with depressive-like behaviors were significantly prevented by chronic D-mannose treatment (Fig. 3E–H, S7A–C). By analyzing the metabolites in the 5-HT biosynthesis pathway, the decrease in the ratio of 5-hydroxytryptophan (HTP) to tryptophan, rather than that of HIAA to 5-HT, was significantly prevented in response to chronic D-mannose feeding, further supporting that the reduction in TPH2 activity in mice with depressive-like behaviors is blocked by oral D-mannose administration (Fig. 3I). When we suppressed brain TPH2 activity by injecting mice with its inhibitor PCPA [29], we found that D-mannose-mediated recovery of hippocampal 5-HT in mice with depressive-like behaviors was abolished, and the antidepressive effect of D-mannose was consequently abrogated, as evidenced by the TST and FST (Fig. 3J–M). These observations indicate that TPH2 is the target of chronic D-mannose administration in treating depression. Moreover, when we downregulated hippocampal ACSS2, the D-mannose-mediated antidepressant effect was abolished as well, suggesting that ACSS2 is required for D-mannose-mediated long-lasting antidepressant function via TPH2 (Fig. 3N–Q). As expected, D-mannose-mediated blockade of decreases in TPH2 and 5-HT in the hippocampus of mice with depressive-like behaviors was accordingly abrogated in response to ACSS2 downregulation (Fig. 3R–T). Altogether, we concluded that the ACSS2-TPH2 axis was required for D-mannose to persistently resist depression.

### ACSS2 promotes *TPH2* histone acetylation and transcription in response to D-mannose-mediated AMPK activation

**Fig. 4:**
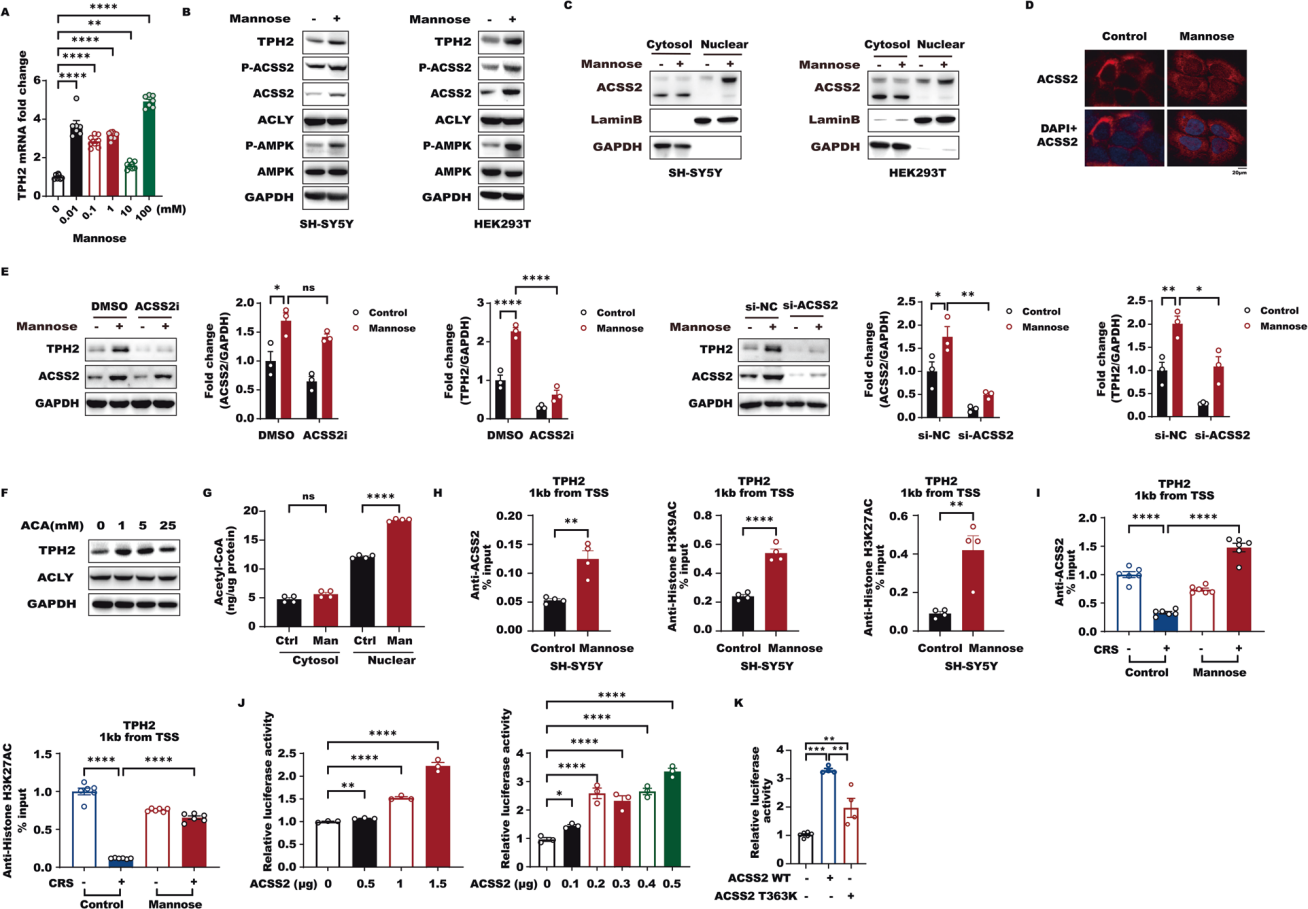
ACSS2 promotes *TPH2* histone acetylation and transcription in response to D-Mannose-mediated AMPK activation. **A** RT-PCR gene expression analysis of *TPH2* in SH-SY5Y cells with the increasing D-Mannose (0–100 mM) for 12 h. Data were normalized with β-actin mRNA levels and presented as fold changes without stimulation. Scale bars represent mean values and error bars represent SEM of triplicate samples. **B** Immunoblot analyses of SH-SY5Y (left) and HEK293T (right) cells expressing TPH2 under its native promoter with or without 1 mM D-Mannose incubation for 24 h with the indicated antibodies were performed. **C** Subcellular fractions of cytoplasm and nuclear were extracted from SH-SY5Y and HEK293T cells with 1 mM D-Mannose for 24 h. Immunoblot experiments were then carried out with indicated antibodies. The signals of GAPDH and LaminB were shown as controls for cytoplasm and nuclear fraction. **D** SH-SY5Y cells were treated with or without 1 mM D-Mannose for 6 h. Immunofluorescence staining of ACSS2 and DAPI was performed. **E** Downregulation of ACSS2 were conducted in SH-SY5Y cells by small RNA interference against ACSS2 or inhibitor (10 μM, 24 h) with 1 mM D-Mannose for 24 h. Immunoblots were performed with the indicated antibodies. **F** SH-SY5Y cells were permeabilized with digitonin (30 μg/mL) for 5 min and treated with the indicated concentrations of acetyl-CoA for 30 min. Immunoblot analyses of TPH2 and ACLY levels were performed. The loading of GAPDH was shown as a control. **G** SH-SY5Y cells were pretreated with 1 mM D-Mannose for 24 h. The levels of acetyl-coa in cytoplasmic and nuclear were measured by ELISA after subcellular fraction of cytoplasm and nuclear extraction. The data are presented as the mean ± SEM from four independent experiments. **H** SH-SY5Y cells were incubated with 1 mM D-Mannose for 24 h. ChIP analyses using an anti-ACSS2, anti-H3K9AC and anti-H3K27AC antibodies were performed. The histogram shows the amount of immunoprecipitated DNA expressed as a percentage of the total input DNA. The data are presented as the mean ± SEM of quadruplicate samples. **I** The Hip tissues from Control, CRS-Control, D-Mannose and CRS-D-Mannose groups were collected and tissues of two male mice per group were added together to be a sample for chromatin immunoprecipitation (CHIP) assay. CHIP analyses using an anti-ACSS2 and anti-H3K27AC antibodies were performed. The histogram shows the amount of immunoprecipitated DNA expressed as a percentage of the total input DNA. The data are presented as the mean ± SEM of six samples of male mice (*n* = 12). **J** HEK293T cells expressing luciferase with human *TPH2* promoter or mouse *tph2* promoter were transfected with increasing ACSS2 expression plasmids. The luciferase activities were examined and data of sample without exogenous ACSS2 expression was normalized to 1. The data are presented as the mean ± SEM from three independent experiments. **K** HEK293T cells transfected with plasmids expressing luciferase under human TPH2 promoter was co-transfected with wild type ACSS2 or T363K mutant plasmid. The luciferase activities were examined and the data of sample without exogenous ACSS2 expression was normalized to 1. The data are presented as the mean ± SEM from three independent experiments.

As we described above, both acute and chronic D-mannose administration induced ACSS2 to activate *TPH2* transcription, thereby inducing fast-acting and long-lasting antidepressant responses. Next, the mechanism of ACSS2 regulation in *TPH2* transcription in response to D-mannose was further investigated. Interestingly, we observed that D-mannose significantly increased *TPH2* expression in a dose-dependent manner in SH-SY5Y human neuroblastoma cells (Fig. 4A, S8A). Correspondingly, TPH2 protein was also elevated in both SH-SY5Y and HEK293T cells transfected with the TPH2 expression plasmid under its native promoter in response to D-mannose along with specific elevation of global ACSS2, rather than ACLY (Fig. 4B). Here, it was of note that doses from 10 μM to 10 mM D-mannose all induced TPH2 transcription, suggesting that physiological concentrations of D-mannose (50 μM–100 μM in blood; 50 μM–70 μM in cerebrospinal fluid) can promote *TPH2* transcription [30, 31]. ACSS2 phosphorylated at serine 659 by AMPK is allowed to enter the nucleus and locally produce acetyl-CoA from acetate for histones, thereby activating target gene transcription [3]. Therefore, we hypothesized that ACSS2 might activate *TPH2* transcription by promoting histone acetylation. As expected, D-mannose activated AMPK and triggered ACSS2 S659 phosphorylation and nuclear translocation without influencing mTOR activity (Fig. 4B–D, S8B). The downregulation of ACSS2 levels or its enzymatic inhibition suppressed D-mannose-mediated expression of *TPH2* (Fig. 4E). In conclusion, AMPK-dependent nuclear ACSS2 is essential for D-mannose to activate *TPH2* transcription.

Nuclear ACSS2 has been reported to supply acetyl-CoA groups for the histones of target genes. Next, we observed that exogenous acetyl-CoA triggered TPH2 expression and that higher nuclear acetyl-CoA levels were generated in response to D-mannose, suggesting that nuclear ACSS2 locally generates acetyl-CoA to contribute to *TPH2* transcription (Fig. 4F, G). Through the use of a chromatin immunoprecipitation (ChIP) assay, we found that ACSS2 bound to the *TPH2* promoter and that D-mannose recruited more ACSS2 to the *TPH2* promoter and thus caused higher degrees of histone H3K9 and H3K27 acetylation in the *TPH2* promoter (Fig. 4H). Consistent with these observations in vitro, in mice with CRS-induced depressive-like behaviors, D-mannose treatment reversed the reductions in ACSS2 in the *TPH2* promoter and hippocampal acetyl-CoA levels caused by CRS in vivo (Fig. 4I, S8C). Importantly, histone H3K27 acetylation of the *TPH2* promoter was downregulated in mice with depressive-like behaviors (Fig. 4I). D-mannose treatment, however, blocked this reduction without influencing total hippocampal histone H3K27 and H3K9 acetylation levels and histone H3 distribution on the *TPH2* promoter (Fig. 4I, S8D, E). To further support these findings, ACSS2 increased both human and mouse *TPH2* promoter activity in a luciferase reporter assay, while the ACSS2 T363K mutant lost the ability to activate the *TPH2* promoter, suggesting that TPH2 promoter activity is dependent on the ACSS2 enzymatic activity of synthesizing acetyl-CoA (Fig. 4J, K). On the basis of these findings, we propose that D-mannose induces ACSS2 nuclear translocation via AMPK to activate *TPH2* histone acetylation and transcription.

### D-mannose activates AMPK via the Ca^2+^-CAMKK2 and lysosomal AXIN-LKB1 pathways to elevate nuclear ACSS2 levels

**Fig. 5:**
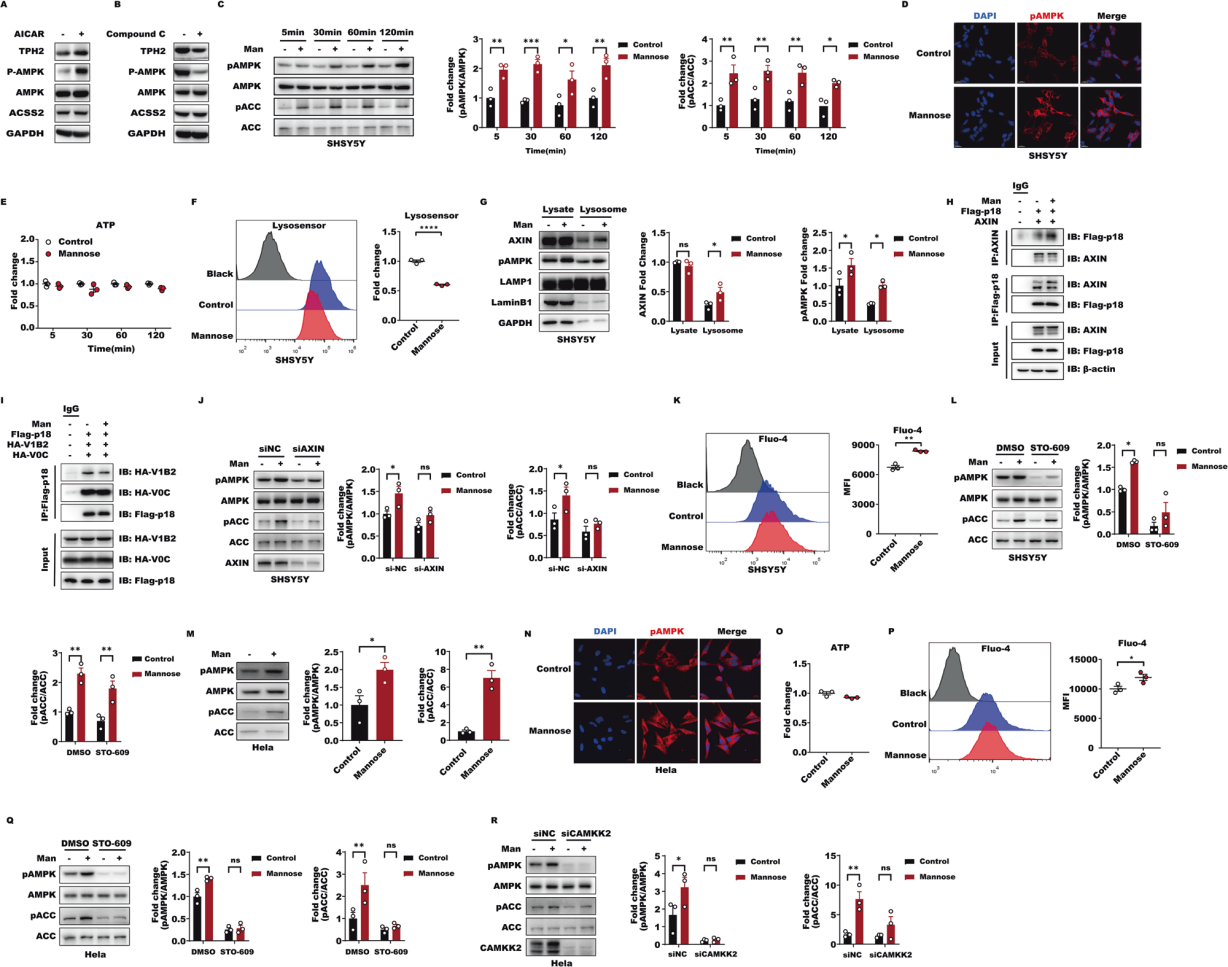
D-Mannose rapidly and persistently activates AMPK via the Ca^2+^-CAMKK2 and lysosomal AXIN-LKB1-v-ATPase pathway to promote nuclear ACSS2-mediated *TPH2* transcription. **A** HEK293T cells expressing TPH2 with its native promoter were incubated by 500 μM AMPK agonist AICAR for 5 h to activate AMPK. Immunoblotting analyses were performed with the indicated antibodies. **B** HEK293T cells expressing TPH2 with its native promoter were incubated by 10 μM AMPK antagonist Compound C for 60 min to inhibit AMPK. Immunoblotting analyses were performed with the indicated antibodies. **C** The lysates of SH-SY5Y at different times with or without 1 mM D-Mannose stimulation were subjected to western blot with indicated antibodies. **D** Representative fluorescent images of pAMPK and DAPI in SH-SY5Y with or without 1 mM D-Mannose stimulation for 30 min. **E** ATP content in SH-SY5Y lysates with or without 1 mM D-Mannose stimulation at different times. **F** Lysosome PH were evaluated by flow cytometry with LysoSensor Green DND-189 (1 uM, 45 min) staining in response to 1 mM D-Mannose for 30 min in SH-SY5Y. **G** The lysates and lysosomal lysates of SH-SY5Y stimulated with or without 1 mM D-Mannose for 30 min were western blotted with the indicated antibodies. **H** CoIP of FLAG-tagged p18 with AXIN in HEK293T cells with or without 1 mM D-Mannose stimulation for 24 h. **I** CoIP of FLAG-tagged p18 with HA-tagged V0C or V1B2 in HEK293T cells with or without 1 mM D-Mannose stimulation for 24 h. **J** The lysates of SH-SY5Y stimulated with or without 1 mM D-Mannose for 30 min after 48H interference with siNC or siAXIN were subjected to western blot with indicated antibodies. **K** Ca2^+^ was assessed by flow cytometry with Fluo-4, AM (5 μM, 30 min) staining in response to 1 mM D-Mannose 30 min in SH-SY5Y. **L** The lysates of SH-SY5Y co-stimulated with or without 1 mM D-Mannose for 30 min following 30 min stimulation with DMSO or 25uM STO-609 were subjected to western blot with indicated antibodies. **M** The lysates of Hela stimulated with or without 1 mM D-Mannose for 30 min were subjected to western blot with indicated antibodies. **N** Representative fluorescent images of pAMPK and DAPI in Hela with or without 1 mM D-Mannose stimulation for 30 min. **O** ATP content in Hela lysates with or without 1 mM D-Mannose stimulation for 30 min. **P** Ca2^+^ was assessed by flow cytometry with Fluo-4, AM (5 μM, 30 min) staining in response to 1 mM D-Mannose for 30 min in Hela. **Q** The lysates of Hela co-stimulated with or without 1 mM D-Mannose for 30 min following 30 min stimulation with DMSO or 25uM STO-609 were subjected to western blot with indicated antibodies. **R** The lysates of Hela stimulated with or without 1 mM D-Mannose for 30 min after 48H interference with siNC or siCAMKK2 were subjected to western blot with indicated antibodies.

We have demonstrated that D-mannose induces ACSS2-dependent *TPH2* histone acetylation and transcription to resist depressive-like behaviors in mice. We hypothesized that, during this process, ACSS2 nuclear translocation, which is dependent on AMPK activation, may be required for TPH2 expression. As expected, when we used AICAR or Compound C to activate or inhibit AMPK, respectively, TPH2 was enhanced and suppressed by AICAR and Compound C inhibition, respectively, indicating that AMPK activation is required for ACSS2-mediated TPH2 transcription (Fig. 5A, B). Considering the fast-acting and long-lasting antidepressant effects of D-mannose, further investigation is needed to determine how AMPK is rapidly and persistently activated to promote nuclear ACSS2 localization. First, we proved that D-mannose rapidly triggered AMPK activation after 5 min of treatment, and this effect lasted for 2 h in SH-SY5Y cells without influencing cellular ATP levels (Fig. 5C–E). This finding promotes us to speculate that D-mannose may activate AMPK via the lysosomal AXIN-LKB1-v-ATPase pathway in an ADP/AMP-independent manner [32]. Next, we evaluated the changes in v-ATPase activity in response to D-mannose and found that D-mannose significantly impaired lysosome acidification and function, as illustrated by LysoSensor staining, suggesting that v-ATPase activity may be suppressed by D-mannose to facilitate AXIN lysosomal translocation (Fig. 5F). Next, we observed that D-mannose recruited more AXIN to the lysosome but suppressed v-ATPase localization on lysosomes along with higher levels of phosphorylated AMPK in the isolated lysosomes (Fig. 5G–I). These findings demonstrate that D-mannose can impair v-ATPase function and thus lysosome acidification to facilitate AXIN lysosome recruitment, thereby causing AMPK activation. However, the downregulation of AXIN partially, rather than completely, abolished D-mannose-mediated AMPK activation, indicating that D-mannose activates AMPK in a manner that is not fully dependent on AXIN (Fig. 5J). The findings of extensive reports support that calcium influx is able to activate CAMKK2 (Camkkβ) to directly trigger AMPK activation [33, 34]. Therefore, we examined cellular calcium changes and found that calcium levels were significantly elevated by D-mannose (Fig. 5K). Accordingly, the CAMKK2 inhibitor STO-609 partially abolished D-mannose-induced rapid AMPK activation (Fig. 5L). These data supported that ACSS2 rapidly and persistently activated AMPK through both the calcium and lysosomal AXIN-LKB1 pathways. To further support Ca^2+^ participation in the D-mannose activation of AMPK, we observed that D-mannose also increased cellular Ca^2+^ levels and thus triggered AMPK activation without influencing cellular ATP levels in HeLa cells, which lack LKB1, and CAMKK2 has been found to be responsible for AMPK activation in an AXIN-LKB1-independent manner [33] (Fig. 5M–P). Correspondingly, the CAMKK2 inhibitor, STO-609, and the downregulation of CAMKK2 completely abrogated D-mannose activation of AMPK (Fig. 5Q, R). In conclusion, D-mannose rapidly and persistently activates AMPK via Ca^2+^-CAMKK2 and the lysosomal AXIN-LKB1 pathway to facilitate ACSS2 nuclear translocation, thereby allowing *TPH2* histone acetylation and transcription.

## Discussion

Here, we established that the metabolic sensor ACSS2 functions as a novel antidepressant target to potentiate both synaptic plasticity by inhibiting eEF2 phosphorylation to promote BDNF levels and serotonin generation by activating TPH2 transcription, thereby mediating fasting-acting and long-lasting antidepressant responses. Extensive studies have been conducted to elucidate the mechanism of depression pathogenesis to explore the available therapeutic treatments. In recent decades, various traditional antidepressants have been developed, but there are still two limitations to current depression treatments, which predominantly focus on targeting deficits in serotonergic and noradrenergic neurotransmission [14–16]. First, a significant delay often occurs at the early stage of treatment, normally from weeks to months. Second, approximately one-third of patients fail to display an adequate response and are subject to developing persistent, treatment-resistant depression (TRD) [35]. Therefore, because there have been no new breakthrough pharmacological treatments for depression by selective serotonin reuptake inhibitors, ketamine, as an uncompetitive N-methyl-D-aspartate (NMDA) glutamate receptor antagonist, is emerging as a novel research hot spot and has thus been extensively investigated for its production of rapid and profound reductions in depressive-like behaviors [7, 13, 15, 36]. Furthermore, ketamine-provoked rapid antidepressant effects have been shown to largely depend on BDNF to improve synaptic impairments in depression [13, 37, 38]. Although ketamine has shown great efficacy in rapid antidepressant action and TRD treatment and there is cautious optimism surrounding the therapeutic potential of ketamine for depression, there is no doubt that the diverse potential and long-term side effects and unsolved clarification of its underlying therapeutic action limit its applications in clinical treatment. Therefore, the identification of novel antidepressant effect targets, especially those that regulate both BDNF-dependent rapid antidepressant action and serotonergic neurotransmission-mediated long-lasting antidepressant effect, is extremely significant for depression prevention and treatment. At this point, ACSS2 was found to control both BDNF- and TPH2-dependent serotonin generation and thus may be a novel target to systematically improve depressive symptoms.

Regarding ACSS2 regulation of BDNF, we support that ACSS2 functions as a novel regulator to inhibit eEF2 phosphorylation, thereby elevating BDNF levels. Ketamine treatment, can rapidly and significantly increase plasma BDNF in treatment responders compared to nonresponders [39]. In preclinical studies, BDNF is also elevated in response to ketamine [13, 40]. Thus, ACSS2 function in plasma BDNF needs to be further evaluated. Similar to ketamine, as a rapid ACSS2 inducer, D-mannose also led to the deactivation of eEF2 phosphorylation to release BDNF, further supporting the rapid antidepressant action of ACSS2. In line with this finding, the downregulation of hippocampal ACSS2 led to BDNF reduction and synaptic formation impairment, while enhanced ACSS2 by D-mannose significantly restored the BDNF decrease and spine formation. Altogether, these findings show that ACSS2 can suppress eEF2 phosphorylation to release BDNF, thereby mediating a rapid antidepressant response.

On the other hand, ACSS2 was identified as a novel coactivator that potentiates *TPH2* histone acetylation and transcription. TPH2 principally determines central 5-HT production and homeostasis, while its dysfunction has been implicated in many neuropsychiatric disorders, including depression [27, 41, 42]. Considering this, TPH2 has been extensively investigated since its identification in 2003. However, it is still unclear how TPH2 responds to cellular metabolism changes with AMPK activation. Here, we show that ACSS2 can sense AMPK activation to translocate into the nucleus and regulate TPH2 histone acetylation and transcription.

To support ACSS2 as a potential therapeutic target, D-mannose was specifically selected as a rapid ACSS2 inducer to mediate fast- and long-acting antidepressant responses. The findings of a previous report support that D-mannose metabolism dysfunction is closely involved in depression patients or mice with depressive-like behaviors and that vitamin B12 can rapidly normalize mannose metabolism to improve chronic stress induced depressive-like behaviors of mice [43]. Unexpectedly, here, we found that a single gavage of 200 μl 20% D-mannose produced significant reductions in depressive symptoms. In addition, acute single D-mannose administration remarkably reversed CRS-induced synaptic impairments via the deactivation of the eEF2-induced BDNF increase. More importantly, acute and chronic D-mannose supplementation also triggered TPH2 histone acetylation and transcription to facilitate the restoration of the CRS-induced 5-HT reduction. Through molecular docking analysis, it was shown that D-mannose itself seems not to directly inhibit HDAC to promote TPH2 histone acetylation, considering that there are very weak interactions between D-mannose and HDAC compared with those of the HDAC inhibitor TSA (Fig. S9A, B). Thus, the antidepressive function of D-mannose is dependent on ACSS2. Taken together, these results proved the anti-depressive efficacy of D-mannose in mice through the upregulation of BDNF and TPH2. Importantly, D-mannose can rapidly and persistently activate AMPK via Ca^2+^-CAMKK2 and the lysosomal AXIN-LKB1 pathway to promote ACSS2 nuclear localization, thereby facilitating *TPH2* transcription. This is thus explainable for TPH2 enhancement by acute D-mannose treatment within a few hours. In addition, Ca^2+^ influx can also promote BDNF synthesis through NMDA receptors [44]. D-mannose, therefore, may promote BDNF elevation via Ca^2+^ influx. Importantly, the biochemical analysis of D-mannose in peripheral tissues, such as heart, liver, and kidney tissues, demonstrated that D-mannose has no obvious toxic effects. D-mannose also had no influence on hippocampal weight, neuronal energy generation, or the redox state. Here, it should be noted that long-term D-mannose overload induces anxiety-like symptoms [45]. Therefore, the amount of D-mannose should be tightly controlled as a potential therapeutic approach for depression. Moreover, many antidepressant factors have rapid effects but do not rapidly act in humans. Further investigations should be carried out to examine the antidepressant effects of D-mannose in patients. Taken together, these findings show that appropriately administered D-mannose might be a safe but high-efficacy, rapid, and long-lasting antidepressant agent through its actions on ACSS2.

Glucose metabolism fuels more than 95% of ATP production for brain development and function [46]. In humans, glucose levels dynamically fluctuate, and low physiological glucose can activate AMPK to reprogram cell metabolism [32]. D-mannose, which has a physiological blood concentration of less than one-fiftieth of that of glucose, can trigger AMPK as well by perturbing glycolysis and enhancing FOA [47, 48]. Neurotransmission, which contains multiple consecutive processes, including neurotransmitter synthesis, storage/release, signaling, reuptake, and degradation, tightly controls neuron function and continuously adapts to metabolic changes caused by glucose fluctuations and D-mannose. In this study, we uncovered a mechanism by which D-mannose favors central BDNF and 5-HT generation, thereby protecting against CRS-induced depressive-like behaviors in mice. This mechanism reveals a novel function of D-mannose in the central nervous system (CNS) to modulate mood and behavior in response to cellular metabolism changes. Glucose intake has been reported to elevate brain 5-HT content by increasing peripheral tryptophan across the blood-brain barrier to supply a substrate for TPH2 [49]. However, the administration of superphysiological amounts of glucose would result in potentially unwanted risks to human health, such as metabolic syndrome, type II diabetes, chronic inflammation/autoimmunity, and even cancer [50, 51]. In this respect, D-mannose is obviously different from glucose and initiates ACSS2 to target *TPH2* transcription rather than blocking CRS-induced brain tryptophan reduction. Importantly, we observed that hippocampal D-mannose was lowered by CRS, suggesting that D-mannose is responsive to CRS and is physiologically involved in stress-related responses. To further support D-mannose function, ACSS2 is upregulated to transcribe *TPH2*, and thus, we propose that D-mannose-induced *TPH2* expression via ACSS2 plays an important physiological function in maintaining central 5-HT homeostasis and serotonergic neuron function in response to cell metabolic reprogramming.

Overall, our findings are novel in that they demonstrate the function of ACSS2 in mediating fast-acting and long-lasting antidepressant responses, which can suppress eEF2 phosphorylation to elevate BDNF and activate TPH2 transcription. *TPH2* is defined as a novel target of AMPK-dependent nuclear ACSS2 to resist depressive-like behaviors with D-mannose supplementation in mice. In addition to the clinical applications of D-mannose in treating cancer, urinary infections, type 1 diabetes, and diabetic wounds, we also support the potential of D-mannose as a therapeutic strategy for depression and other neuropsychiatric disorders through its actions on ACSS2.

## Materials and methods

### Mice

Six to eight-week-old male or female mice were used in all experiments. The mice were housed in specific pathogen-free conditions on a 12 h light/dark cycle at 18–22 with food and water available *ad libitum* unless notes otherwise. All animal experiments were in accordance with the National Institutes of Health Guide for the Care and Use of Laboratory Animals and were approved by the Animal Care and Utilization Committee of Shandong University. *Acss2* knockout mice were generated using the CRISPR-Cas9 system by GemPharmatech Company. The genotype of mice was determined by DNA sequencing and PCR. The primers used for genotyping were as follows: Primer1-forward: 5′-TCACTTGAGAACTTCCTACCTTAGCC-3′; Primer1-reversed: 5′- GGAAGCAGAATGAGCTGTTAGTGAAAC-3′; Primer2-forward:5′-AAAGCTGTGAAGGTTGACATGCAC-3′, Primer2 -reversed: 5′ATA

TTGATTGGGCTCCCCAGTCT-3′.

Mice were divided randomly into stress groups with or without D-mannose and home cage controls with or without D-mannose. For long-term antidepressant mice, in CRS period, mice were fed with water or 10% D-mannose drinking water for 4 weeks. For the rapid antidepressant mice, 4 weeks after CRS modeling, mice were given 10% D-mannose or water (400 ul/20 g body weight) by gavage, and behavioral tests were performed 3 h after gavage. To block TPH2 activity, we intraperitoneally injected mice with 500 mg/kg PCPA or saline for four consecutive days and the behavioral experiments were performed at 2 h after the last injection.

### Cells

SH-SY5Y human neuroblastoma cells were grown in 1:1 mixture of MEM and F12, supplemented with 10% heat-inactivated fetal bovine serum (FBS), 1 mM sodium pyruvate, 0.1 mM nonessential amino acid, 1.5 g/L sodium bicarbonate, 100 units/mL penicillin, and 100 μg/mL streptomycin. All media and supplements were purchased from Gibco (Gaithersburg, MD, USA). Cells were maintained at 37 °C in a humidified atmosphere of 5% CO2. Cells were treated with D-mannose for the 12 h before RNA or 24 h before protein extraction.

HEK293T cell line was purchased from Shanghai Cell Bank of Chinese Academy of Sciences (GNHu17) and grown in DMEM medium (Gibco, 12100046) supplemented with 10% heat-inactivated fetal bovine serum (FBS; Gibco, 10099–141). Cells were all cultured in a humidified cell incubator with an atmosphere of 5% CO2 at 37 °C.

Hela cell line was purchased from ATCC (American Type Culture Collection) and grown in DMEM medium (Gibco, 12100046) supplemented with 10% heat-inactivated fetal bovine serum (FBS; Gibco, 10099–141). Cells were all cultured in a humidified cell incubator with an atmosphere of 5% CO2 at 37 °C.

### Bacteria

*Escherichia coli* DH5α was cultured in LB medium at 37 °C with 200 rpm/min and used for recombinant plasmid cloning.

## Method details

### HPLC assay for D-mannose

To measure hippocampus D-mannose content, a Develosil ODS-UG-3 column (7.5 cm × 4.6 mm i.d.) from Nomura Chemical Co. Ltd. (Seto, Japan) was utilized in conjunction with HPLC system (LC-20AT) [52]. The mannose concentration was calculated by comparing the peak area of ABEE-labeled mannose with that of ABEE-labeled lactose.

### Chronic restraint stress

To generate the CRS-induced depressive-like behaviors of mice [53], we performed chronic restraint stress with mice daily during 9:00 a.m.–11:00 a.m. for 4 weeks using the well-ventilated polypropylene restrainers in deprivation of food and water. At the end of the stress session, mice were returned to the home cage. Then, mice behavior was evaluated by one behavioral testing (TST, FST, and SPT) per day after the last stressor during the light phase of the cycle between 9:00 a.m. and 4:00 p.m. Before test, mice were allowed 2 h to habituate to the testing rooms.

### Chronic unpredictable mild stress

Male or female C57BL/6J mice were exposed to a variable sequence of chronic unpredictable mild stressors, including food or water deprivation (24 h), overnight illumination, cold on ice (5 min), wet bedding (24 h), titled cage (24 h), tail clip (1 min), physical restraint (2 h), foot shock (30 min), noise (2 h), and shaking (1 h). Each stressor was randomly applied daily for 4 weeks. Behavioral tests were then used to assess anxiety- and depressive-like behaviors in these animals.

#### Tail suspension test

Each mouse was taped with tail (1 cm from tip) and hung to a grid bar over 30-cm height from the ground. Then we recorded the immobile time of the testing mice within 6 min. Immobility was defined as the absence of escape-orientated movement.

#### Forced swimming test

To assess depressive-like behavior, mice were placed into a glass cylinder (25 cm height, 10 cm diameter) filled with water (22 °C) up to a height of 18 cm as previously described earlier [54]. A testing period was defined as 6 min to determine the percentage of time spent immobile [55]. Immobility was defined as being stationary with only enough motion of the tail or forepaws to keep the head above water. When mice used forepaws to move and swim in the center or along the sides of the cylinder, we stopped recording the immobility time. Eventually, we calculated the total immobility time of mice during the 6 min.

#### Sucrose preference test

Sucrose preference procedure was performed as described previously [53, 56]. Mice were habituated to sucrose for 3 days by replacing water bottles with bottles containing sucrose solution (1%). Then, mice were deprived of water for 23 h before getting free access to two bottles, one containing tap water and the other containing 1% sucrose solution, the weights of which were recorded. After 1 h, the weights of bottles were measured again and thus fluid consumption was calculated. Sucrose preference was determined as follows: Sucrose preference (%) = sucrose intake/(sucrose intake + water intake) × 100. Sucrose preference was assessed for two consecutive days. The position of the sucrose and water bottles was alternated daily to avoid spurious effects from a side bias.

#### Open-field test

The OFT apparatus consists of 40 × 40 × 40.5 cm arenas. The individual mouse was placed in the center of the arenas for 10 min. By using the Smart Video Tracking System (Smart 10.0, Panlab, DC, USA), we analyzed the total distance traveled and time spent in the central area of mice.

#### Elevated plus maze

The crossed maze was elevated 50 cm above the floor, with two open arms and two closed arms (30 × 5 × 10 cm, 0.5 cm thick walls). The arms were interconnected by a central platform. Mouse was individually placed in the central platform and allowed to explore for 5 min. We used the video tracking system (Smart 10.0, Panlab, DC, US) to record mice movement. The time spent in the open arms and the number of open arm entries were recorded and analyzed.

### Viral injection

For hippocampal *ACSS2* RNA interference, we injected 2 μl of virus liquid carrying small interfering RNA targeting *acss2* with U6 promoter into the hippocampus by Hamilton micro-syringe with a microinjection pump and an additional 5 min was allowed for diffusion before the microinjection pump was removed. The coordinates were as follows, vHIP: anteroposterior (AP), −2.54 mm; lateral (L), ±2.75 mm; dorsoventral (V), −2.0 mm. Infection effects were evaluated by hippocampal ACSS2 change by western blot. Mice were anesthetized by 5% chloral hydrate (7.5 ml/kg, i.p.) and prepared for virus liquid injection into the hippocampus by Hamilton micro-syringe with a microinjection pump (KDS 200, KD Scientific). The siRNA sequence for ACSS2 was: 5′-CAGGATTGATGACATGCTCAA-3′.

Lentivirus of siACSS2 and siNC (negative control) were packaged and purified separately by Shanghai Genechem Co., Ltd (Shanghai, China).

### Golgi staining

The mice were anesthetized by 5% chloral hydrate (7.5 ml/kg, i.p), and fresh brain tissue were obtained and washed with PBS. All the steps were followed on the manufacture instruction of TM FD Rapid GolgiStain Kit (FD NeuroTechnologies, MD, USA). Z-stack pictures (40×) were captured by Panoramic digital slice scanning microscope (VS120, OLYMPUS, Janpan). The number of nerve dendritic spines per 10 microns was counted.

### In vivo imaging assay

The mice were anesthetized 30 min before the in vivo observation. In order to eliminate the blocking effect of black hair on red fluorescence, the hairs of mice were removed with shaver. Meanwhile, mice front and rear limbs wiped with 75% ethanol to prevent the spontaneous fluorescence. 125 ul 5 mM FITC-Mannose was injected into mice through the tail vein. After 5 min, 30 min, 60 min, 3 h, 6 h, 9 h, 12 h, and 24 h intravenous administration, the pictures of the distribution of red fluorescence in mice was captured by IVIS Lumina II vivo imaging system (PerkinElmer, MA, USA) at Translational Medicine Center Facility of Shandong University.

### ChIP assay

ChIP was performed using a SimpleChIP plus Sonication Chromatin IP kit. Chromatin was prepared from cells in a 10-cm dish was used to determine total DNA input and for overnight incubation with specific antibodies or normal mouse IgG. For mouse hippocampus CHIP, two mice hippocampus were combined (about 30 mg) to extract chromatin. Primer sequences used for PCR are listed in Table S2.

### Enzyme-linked immunosorbent assay (ELISA)

The levels of 5-HT, DA, BDNF, and NE in the hippocampus were measured according to the manufacturer’s protocol by using commercial ELISA kits (Jianglai, Shanghai, China).

For acetyl-CoA level assay, SH-SY5Y cells were prepared for cytoplasm and nuclear isolation and 50 μL subcellular isolates were used to evaluate acetyl-CoA content by ELISA kit according to the manufacturer’s instruction. Light absorbance was read with a multi-mode plate reader (Synergy HT, BioTek Instruments, Inc.) at 405 nm.

### HPLC for 5-HT pathway

Mice hippocampus samples were prepared as described above and each sample was injected onto Kinetex column (2.6μ C18 100 × 4.6 mm) in HPLC system [57]. The chromatographic conditions were the same as that of analyzing tryptophan and kynurenine. Tryptophan, HTP, 5-HT and 5-HIAA were monitored by the fluorescence detector with excitation wavelength of 290 nm and emission wavelength of 330 nm. HTP, 5-HT, tryptophan and 5-HIAA were sequentially separated at 3.2 min, 3.5 min, 5.0 min and 8.4 min and their concentrations were calculated based on standards samples.

### Plasmid construction

The plasmid pCMV-ACSS2 encodes full-length human ACSS2 (NM_018677.4). The mouse or human TPH2 promoter sequences were subcloned to pGL3 basic (Promega, Madison, MI, USA) to drive luciferase expression. TPH2 expressing plasmid under its native promoters were generated by inserting their promoter and coding sequences into pGL3 basic followed by deleting luciferase coding region.

### Immunofluorescence

Cells were seeded on glass coverslips and cultured in DMEM with 10% serum and then washed with PBS followed by being fixed in the stationary liquid from Beyotime Biotechnology (Shanghai, China) for 10 min. They were blocked with 2% BSA for 60 min and stained with primary antibodies overnight at 4 °C. After PBS buffer washing, cells were incubated with horseradish peroxidase conjugated secondary antibody for 1 h. The fluorescence of antibody signals was visualized using Alexa Fluor 594-conjugated IgG (Abcam). DAPI (Invitrogen) was used to stain the nucleus for 10 min. Images were captured by a confocal laser scanning microscope (Carl Zeiss, LSM780, Oberkochen, Germany) with a 63x Plan-Apochromat objective and were analyzed using ZEN lite 2012 software package.

### Western blotting and Co-immunoprecipitation

Cells were lysed with RIPA cell lysis buffer with 1% protease inhibitor cocktail. The supernatant was collected after high-speed (12,000 × *g*) centrifugation for 30 min at 4 °C and protein concentration was determined using a BCA method. All protein signals were collected with different exposure time to make sure the bands were not overexposed and within the linear range to perform quantitative analysis. The band intensity was quantified using the Image J software.

The Nuclear/Cytosol Fractionation Kit was used to extract the cytoplasmic and nuclear proteins from HEK293T and SH-SY5Y cells. The cytoplasmic and nuclear extraction were prepared for acetyl-CoA or western blot analysis.

HEK-293 T cells were lysed by IP buffer. Cell extracts were clarified by centrifugation at 13,400 g, and the supernatants were subjected to immunoprecipitation with the indicated antibodies. After overnight incubation at 4 °C, protein A or G agarose beads were added and left for an additional 3 hr with rotation. Then the protein complexes were washed five times with IP buffer and then subjected to immunoblot analyses with corresponding antibodies as described previously.

### Quantitative real-time polymerase chain reaction (RT-PCR)

Total RNAs were extracted with TRNzol reagent according to the manufacturer’s instructions (Tiangen, Beijing, China) and reversely transcribed into cDNA with PrimeScript™ RT reagent Kit with gDNA Eraser (TAKARA, Japan). The expressions of genes were detected by quantitative RT-PCR (qRT-PCR) using FastStart Universal SYBR Green Master (Roche Applied Science, Penzberg, Germany) on the Bio-Rad CFX 96 (Bio-Rad, California, USA). Cycle threshold (Ct)values were recorded. Data was normalized using β-actin and transformed using the 2^−ΔΔCT^method. The primer sequences are shown in Supplementary Table S2.

### RNA interference and plasmid transfection

HEK293T cell lines with TPH2 expression plasmids under its native promoter in the presence of D-mannose, acetate, propionate, or butyrate were grown in DMEM with 10% serum. ACSS2, control siRNA oligos or plasmids were transfected to cells by using Lipofectamine 2000 (Invitrogen, 11668019) according to the manufacturer’s protocol. Cells were either subjected to immunofluorescence analysis or lysed for SDS-PAGE and western blot analysis.

### Luciferase assay

Luciferase activity was measured with a dual luciferase assay system. The Luciferase reporter vector PGL3 basic and its derivatives with or without human (−1500–+660, 2160 bp) and mouse (−2000–0, 2000bp, mouse) *TPH2* promoter region were transiently transfected into the HEK293T cells in the presence of ACSS2. Twenty-four hours after transfection, Luciferase activity was measured with a dual luciferase assay system (Cat#DL101-01, Vazyme)and the readout was determined using a microplate luminometer (Centro LB 960; Berthold, Wildbad, Germany). Data were analyzed by GraphPad Prism 8. Three independent experiments have carried out for biological replicates.

### Flow cytometry

To assess D-mannose absorption in neuron, FITC labeled D-mannose (Qiyuebio, 20 μM, Ex = 495 nm, Em = 519 nm) and CY5 labeled D-glucose (Qiyuebio, 20 μM, Ex = 650 nm, Em = 670 nm) were utilized and flow cytometry was performed to monitor D-mannose and D-glucose fluorescent signals in SH-SY5Y by CytoFLEX S (BECKMAN COULTER, USA). Followed by D-mannose treatment, SH-SY5Y cells were incubated with a mitochondrial stain (Mito Tracker Red FM, 50 nM, Ex = 644 nm, Em = 665 nm) at 37 °C for 30 min and further analyzed by flow cytometry to evaluate mitochondria changes.

### Molecular docking

To assess the possibility of D-mannose in blocking HDAC activity through physical interaction with HDAC, studies to predict the interaction of D-mannose with HDAC1 was carried out using molecular modeling by AutoDock Tools and Vina 4.2 software [58]. The docked complex was analyzed using BIOVIA Discovery Studio Visualizer to calculate the interaction energy and show the type of interactions between D-mannose and HDAC1.

## Quantification and statistical analysis

### Quantification analysis

The specific western band signals were quantificationally analyzed by Image J. All image statistical analyses were performed using Image Pro-Plus Software and Zeiss Auto-measure software. Nuclear ranges were circled, and the fluorescence intensity of each pixel was calculated by Zeiss Auto-measure software in FRET experiment. More than 50 cells were calculated.

### Statistical analysis

Mean values, standard error of mean, and statistical significance were analyzed by GraphPad Prism 8. Differences between groups were analyzed with the Student’s *t* test (unpaired), one-way or two-way ANOVA followed by the Tukey’s test (for multiple comparisons). *P* values < 0.05 was considered to be statistically significant; *P < 0.05, **P < 0.01, ***P < 0.001, ****P < 0.0001. Results are presented, followed by at least three independent experiments of biological replicates.

### Supplementary information

Figure S1

Figure S2

Figure S3

Figure S4

Figure S5

Figure S6

Figure S7

Figure S8

Figure S9

supplementary figure legends

TableS1-KEY RESOURCES TABLE

Table S2-RT-PCR plasmid CHIP relative primers

## Data Availability

Data from this study are available on request.
