# Supplementary figures and images for: D-mannose is a rapid inducer of ACSS2 to trigger rapid and long-lasting antidepressant responses through augmenting BDNF and TPH2 levels
Source: Transl Psychiatry. 2023 Nov 1;13:338. doi: 10.1038/s41398-023-02636-7 (PMC10620401; doi:10.1038/s41398-023-02636-7)

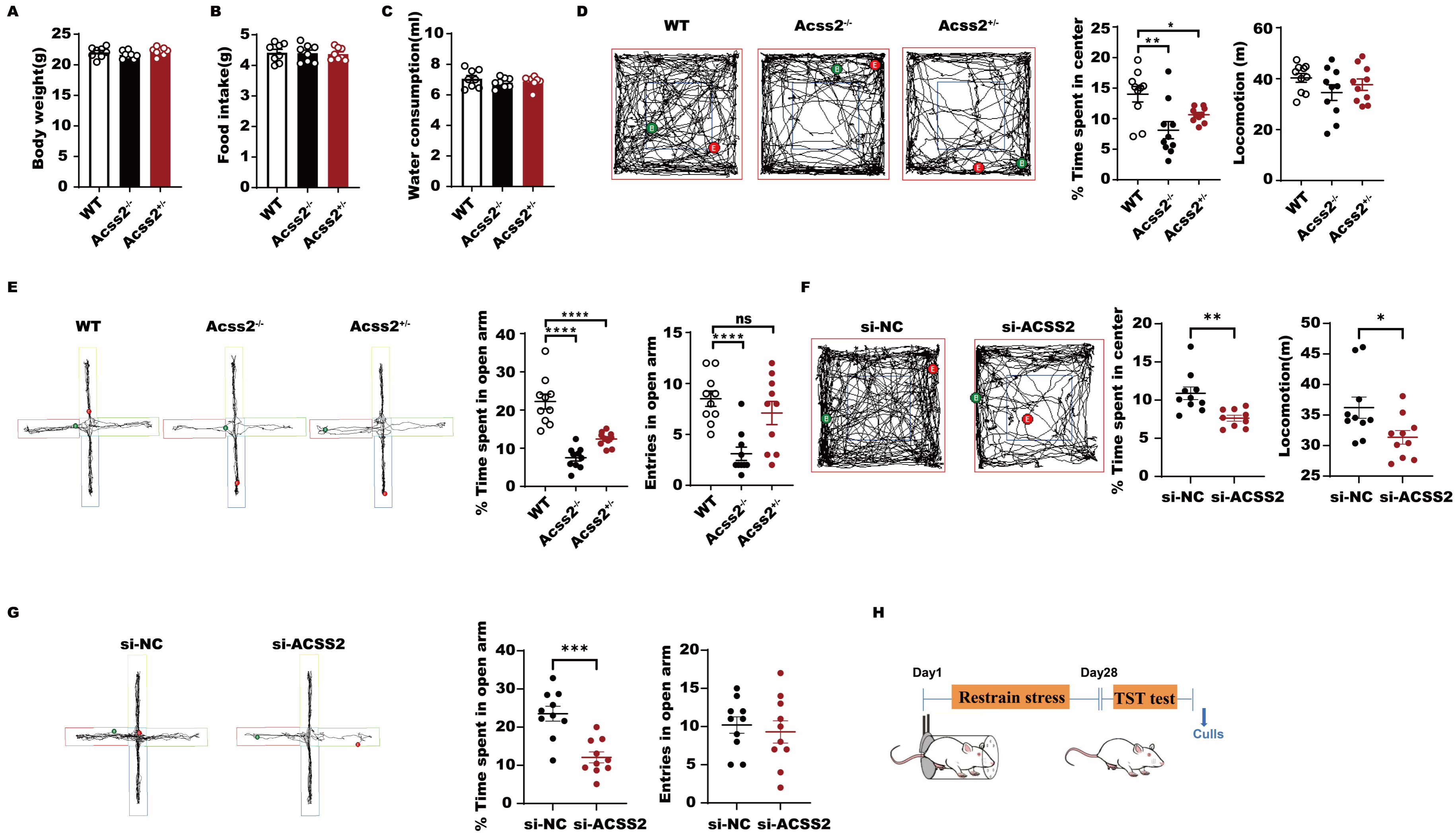

Supplement: Supplementary file 1 — Figure S1 [file 41398_2023_2636_MOESM1_ESM.pdf]

**A**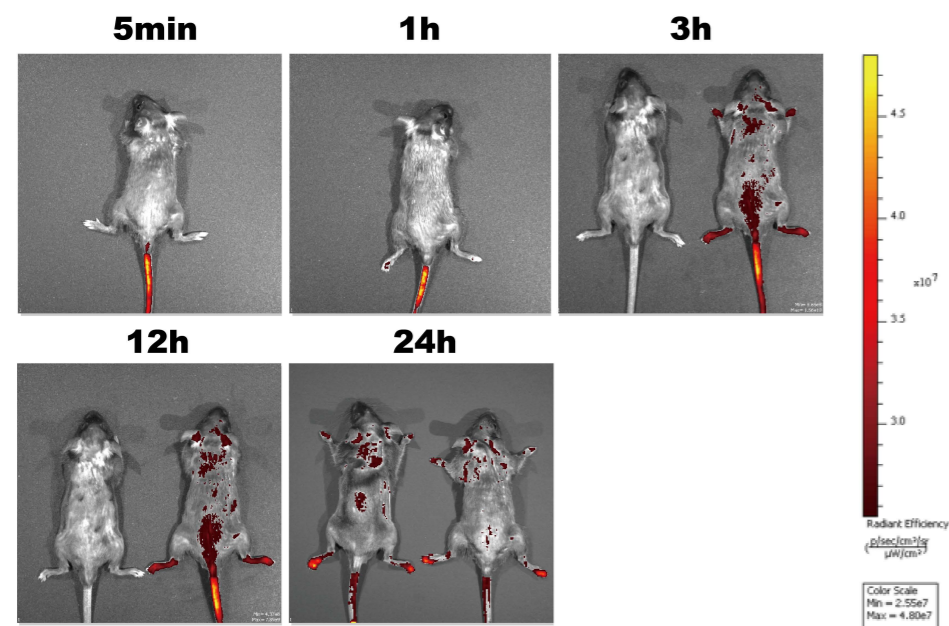**B**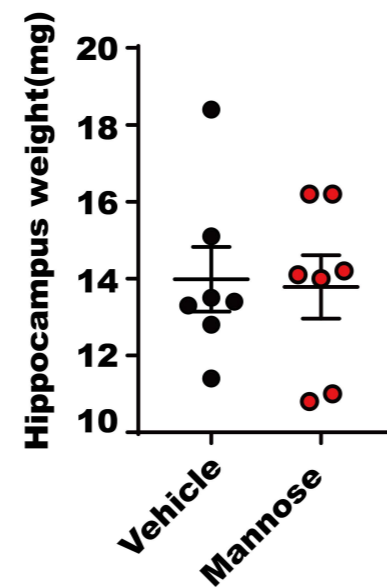**C**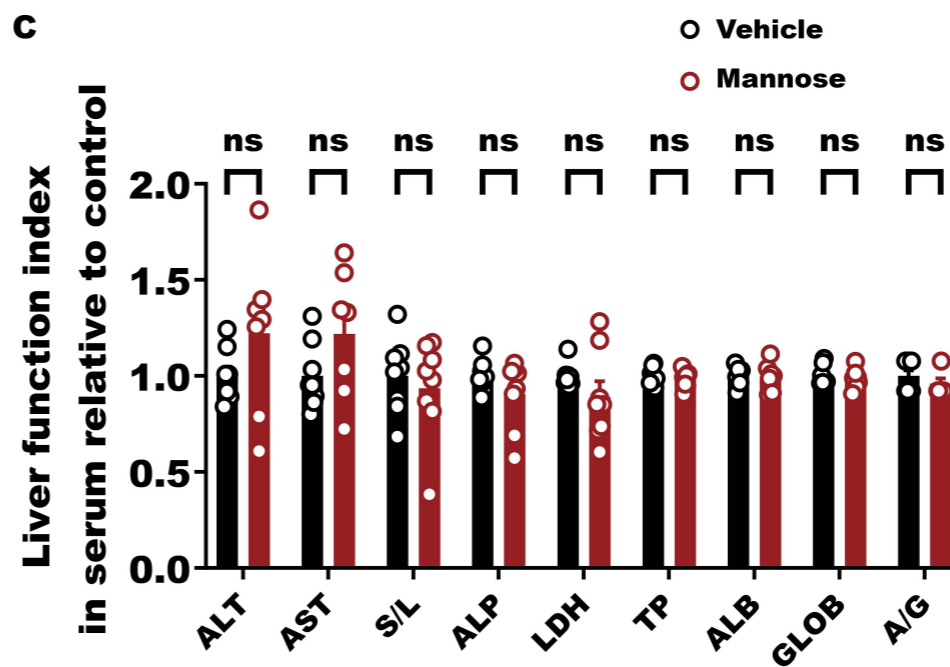**D**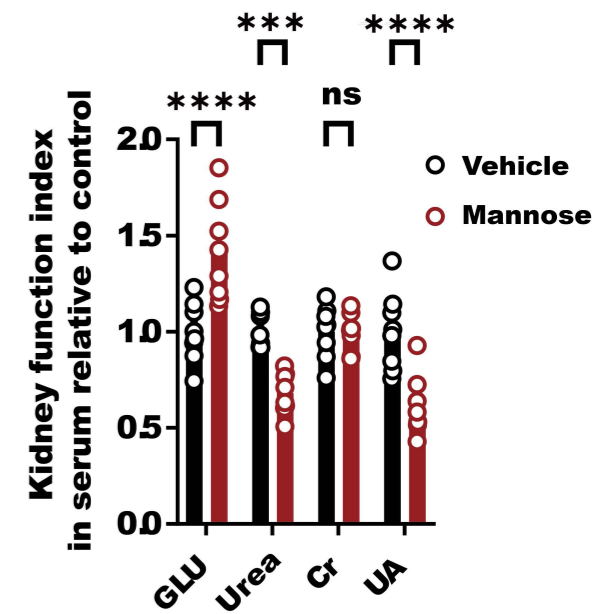**E**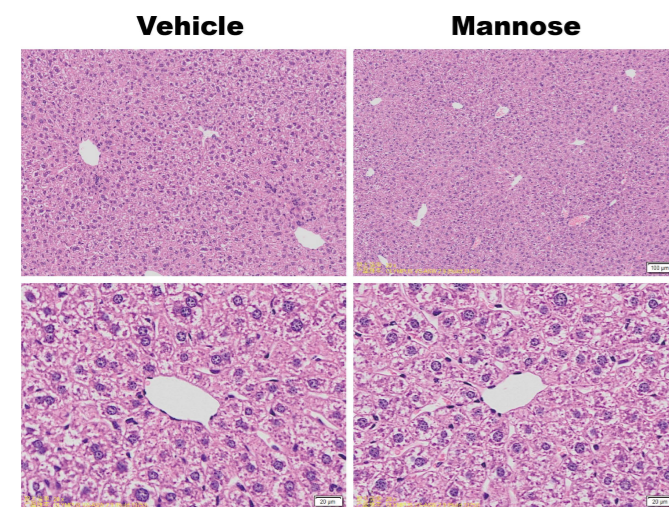**F**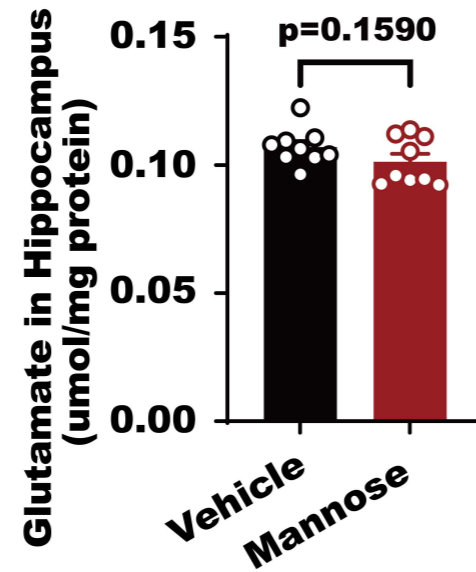**G**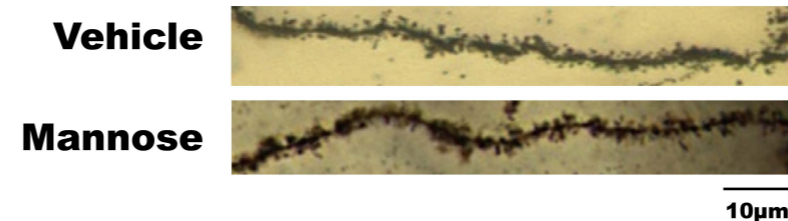

Spine density(spines/10μm)

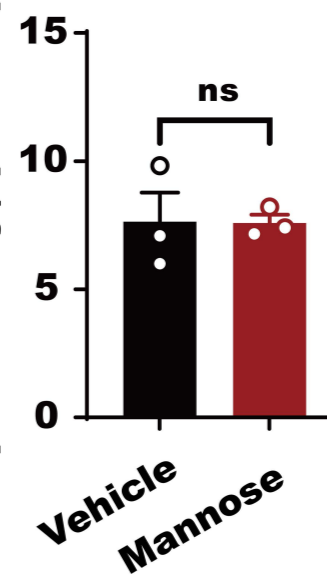

Supplement: Supplementary file 2 — Figure S2 [file 41398_2023_2636_MOESM2_ESM.pdf]

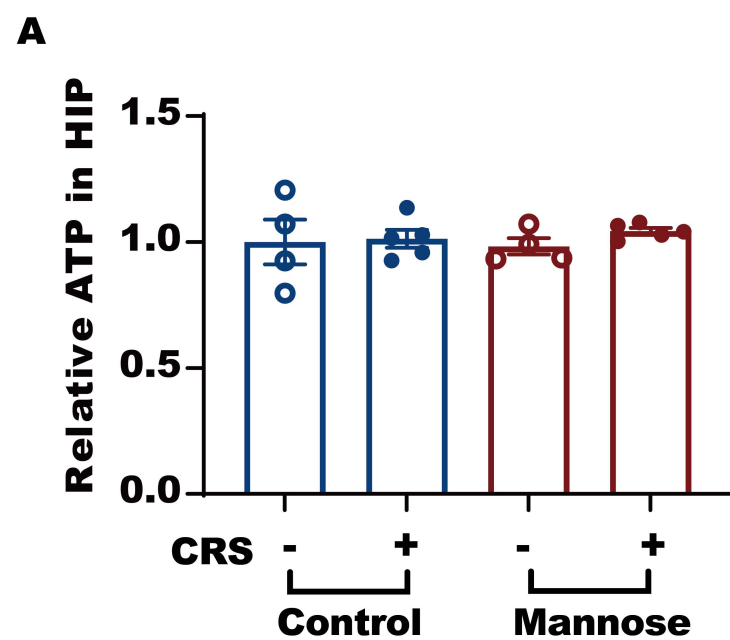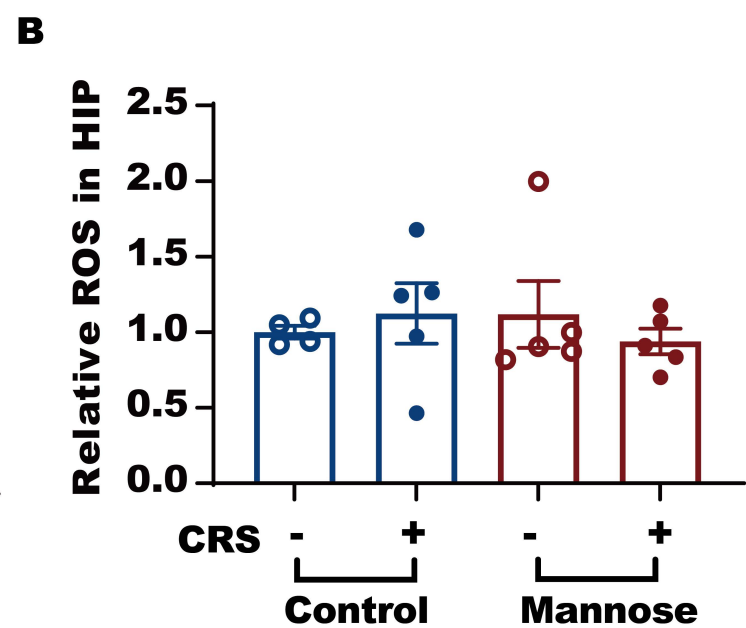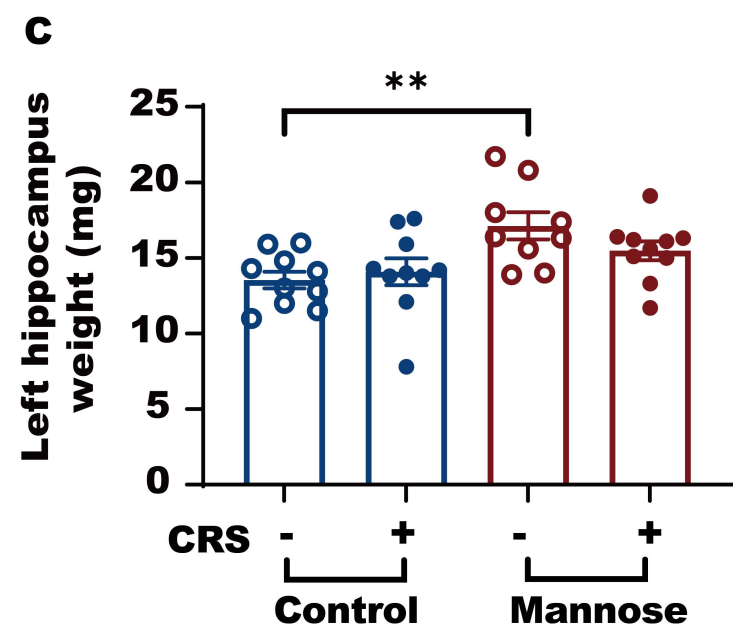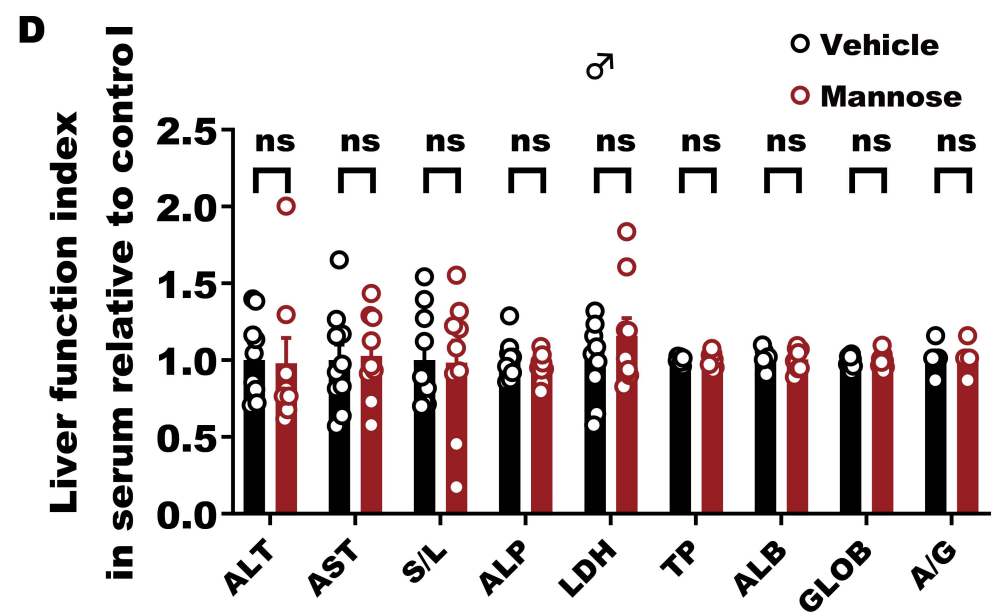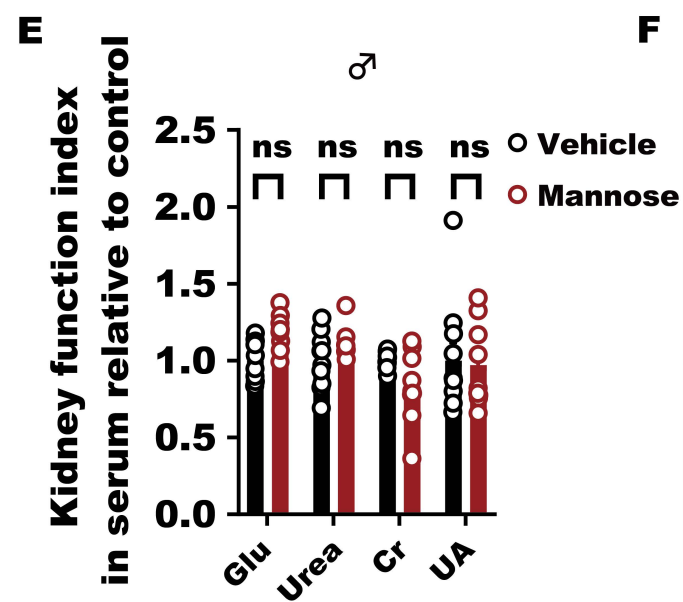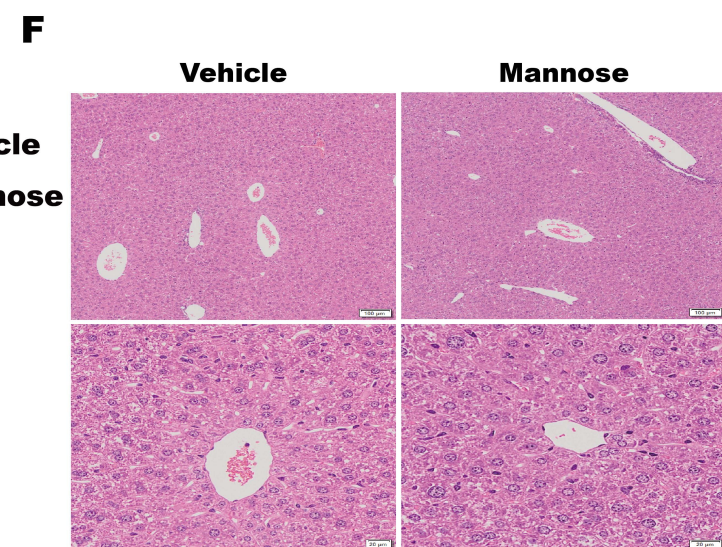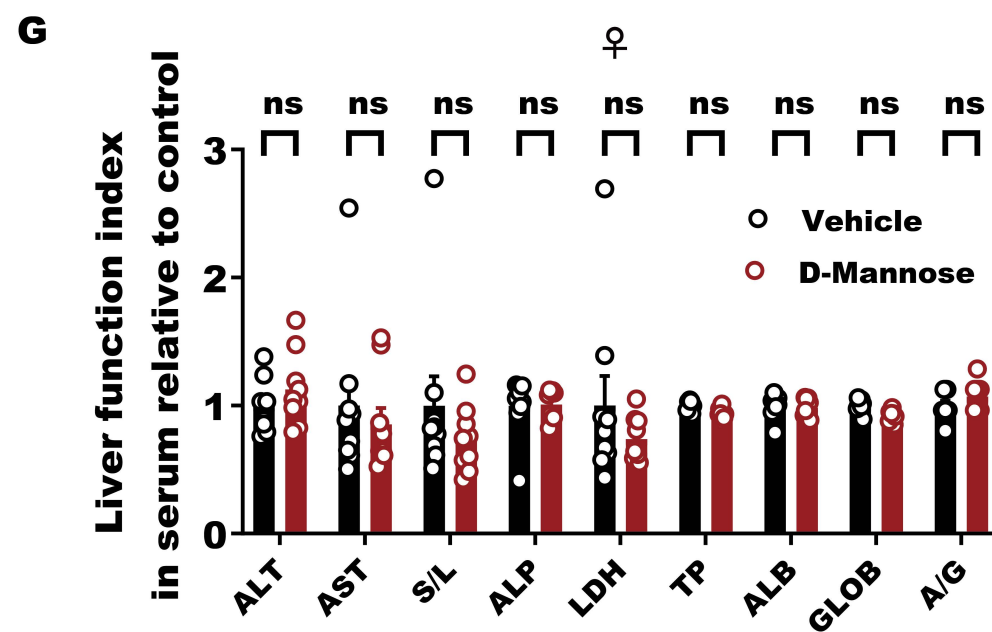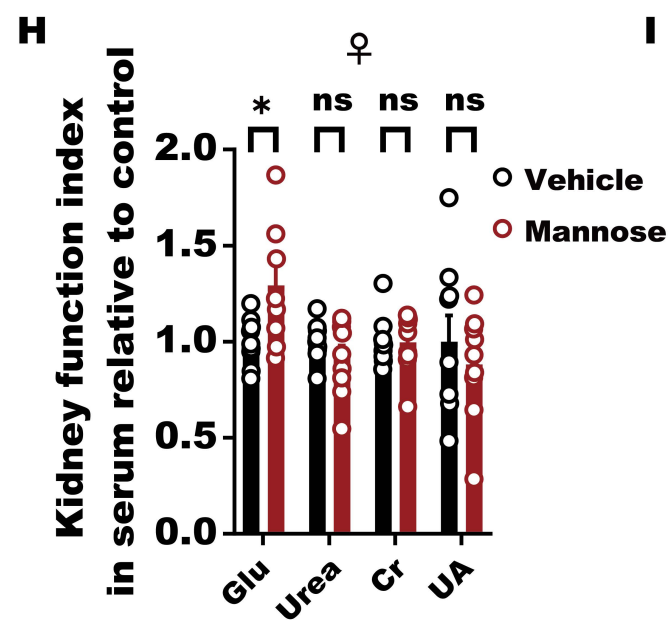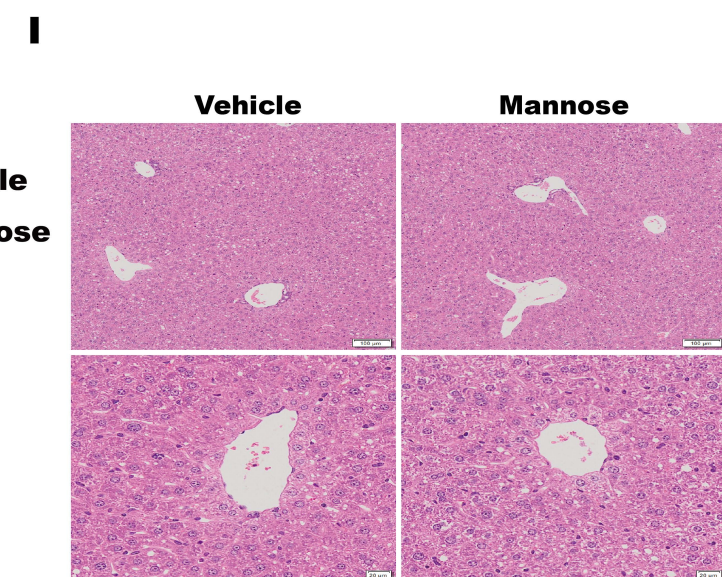

Supplement: Supplementary file 3 — Figure S3 [file 41398_2023_2636_MOESM3_ESM.pdf]

**A**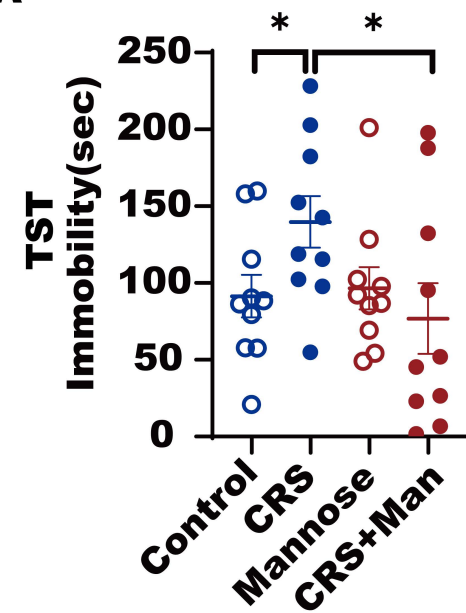**B**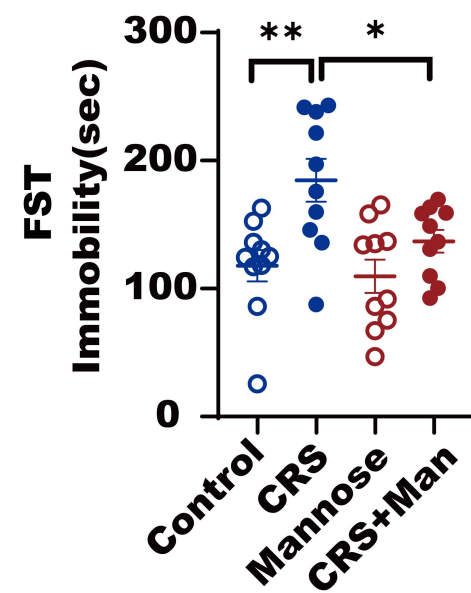**C**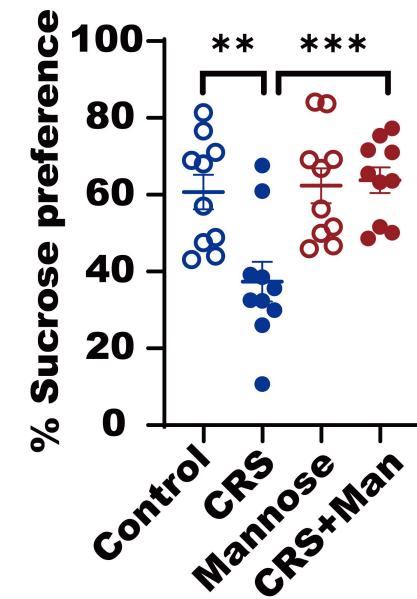**D**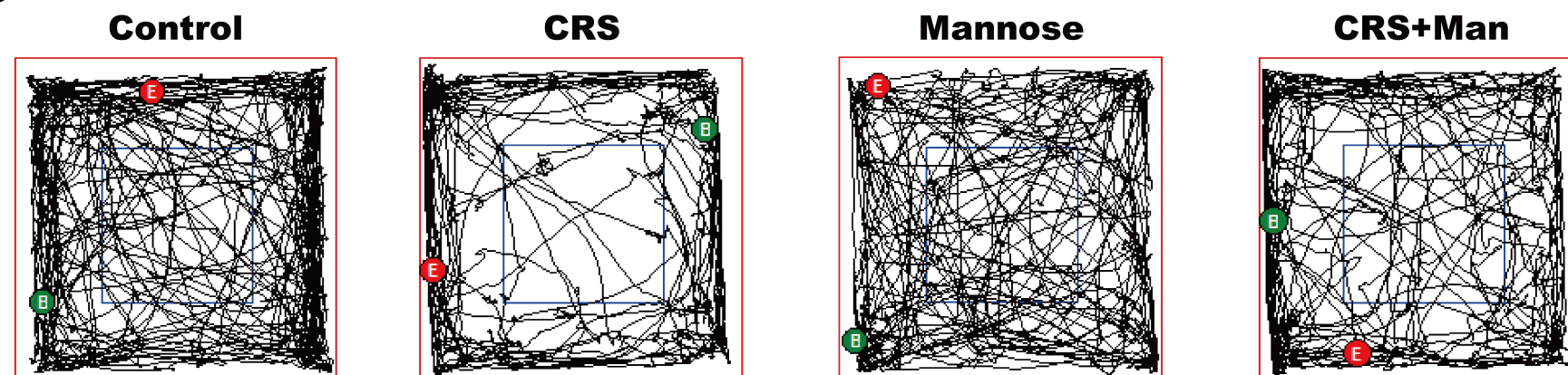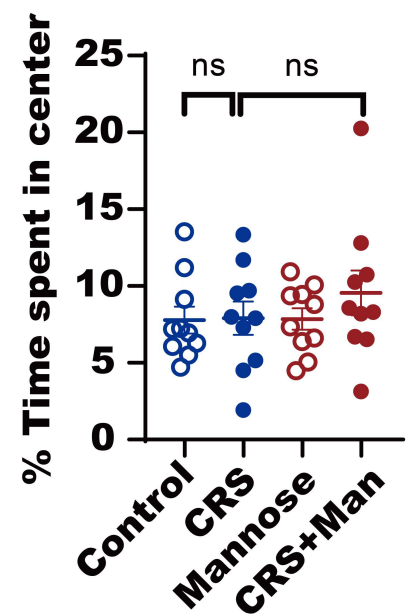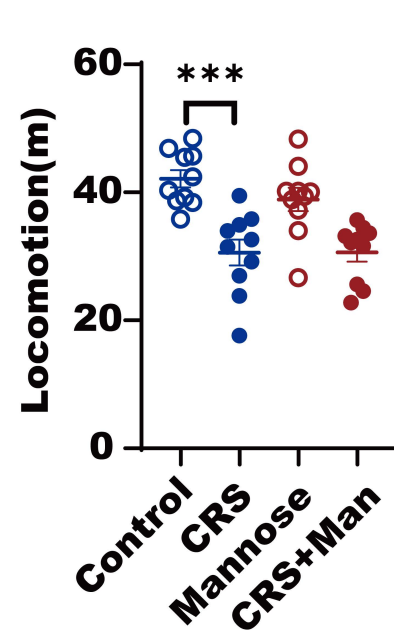**E**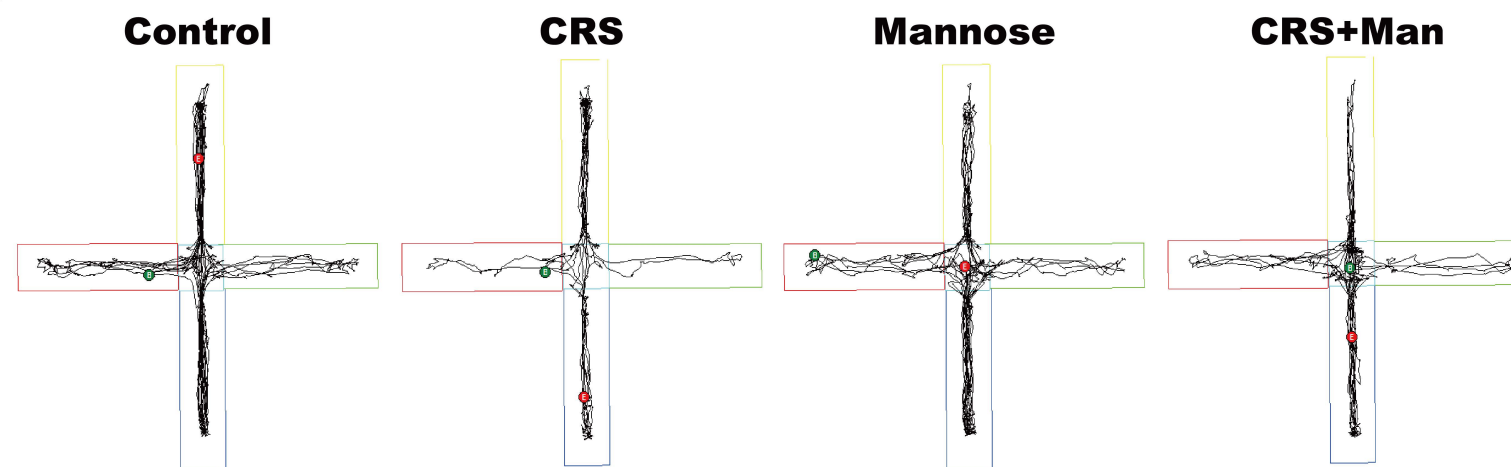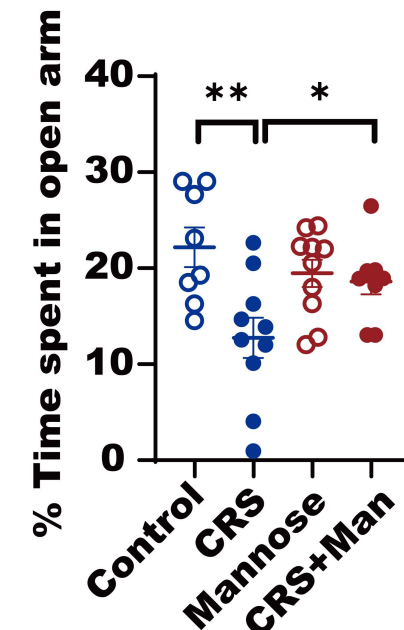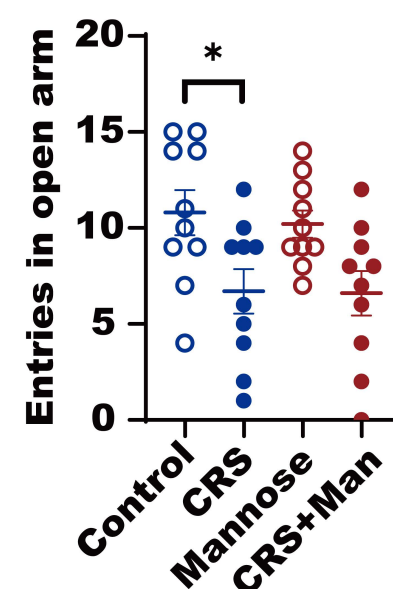

Supplement: Supplementary file 4 — Figure S4 [file 41398_2023_2636_MOESM4_ESM.pdf]

**A**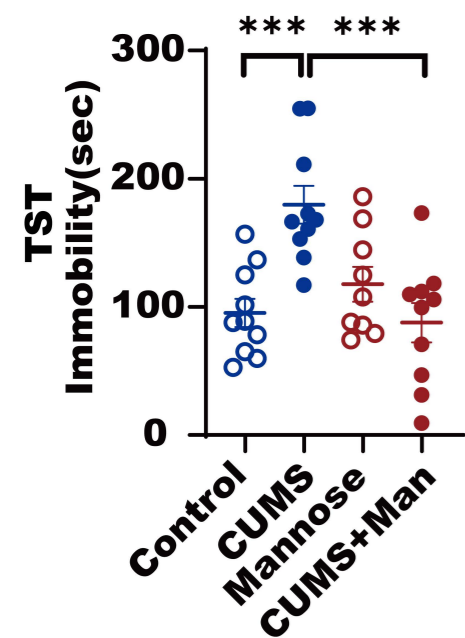**B**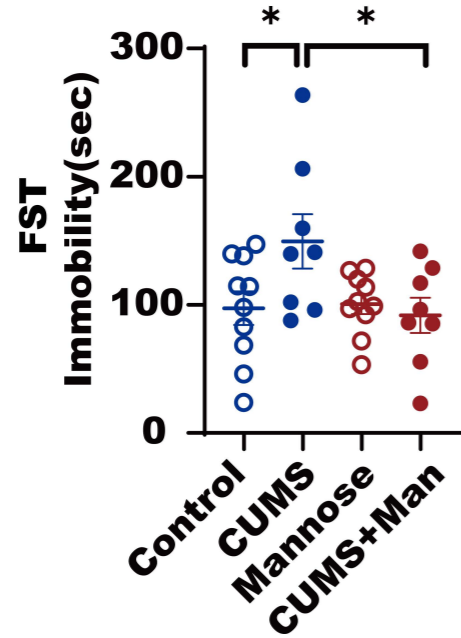**C**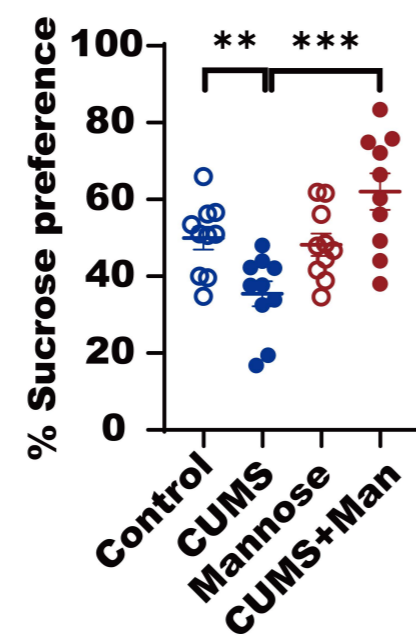**D**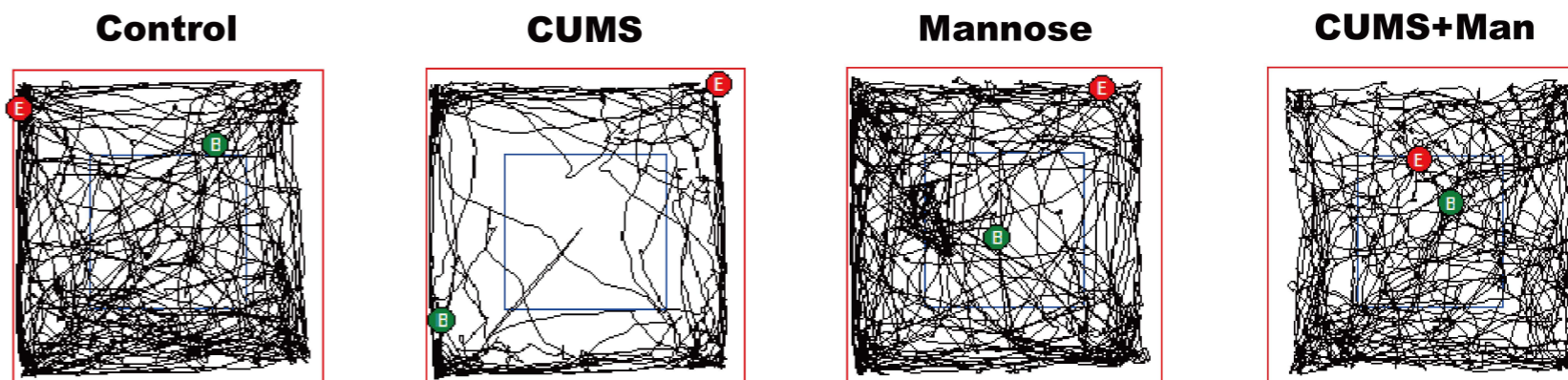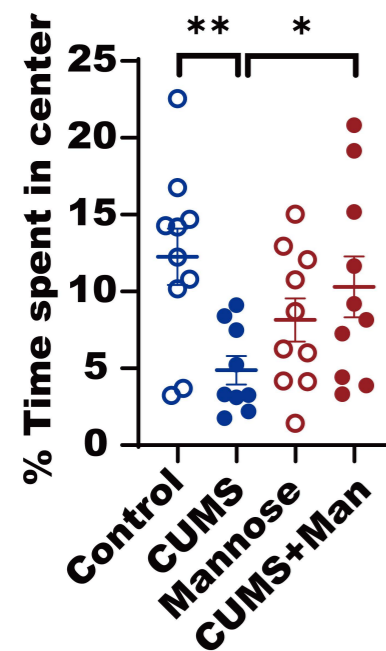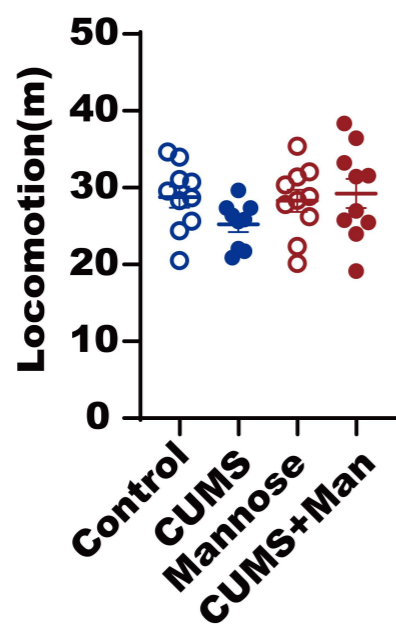**E**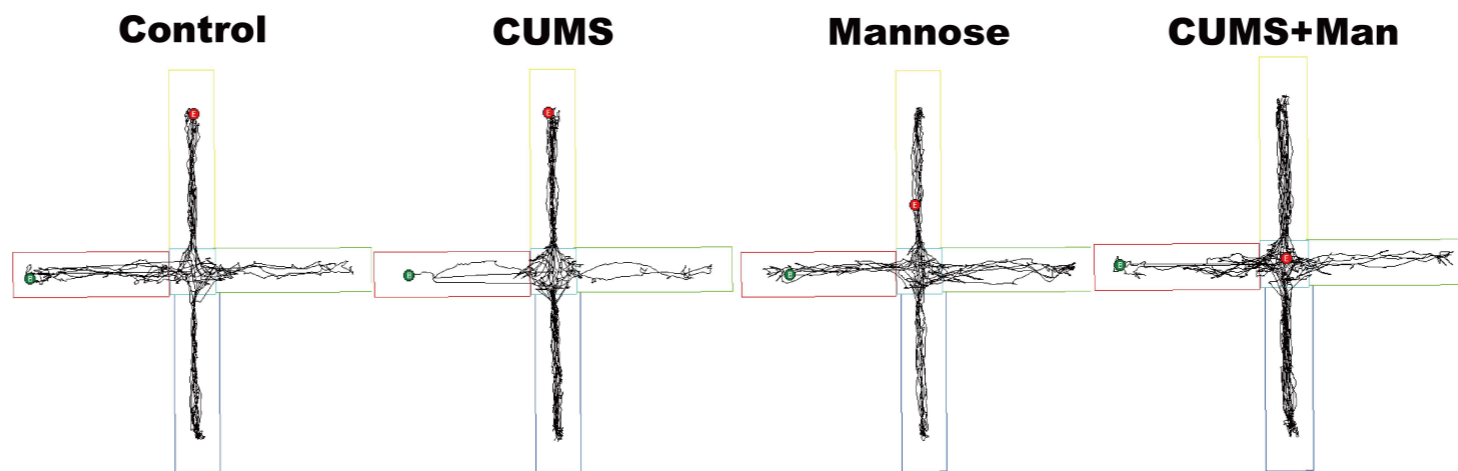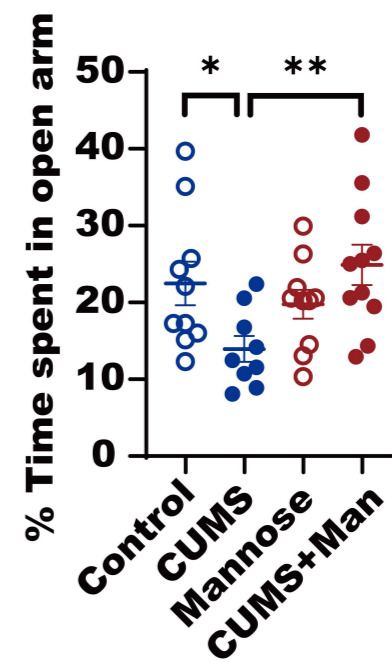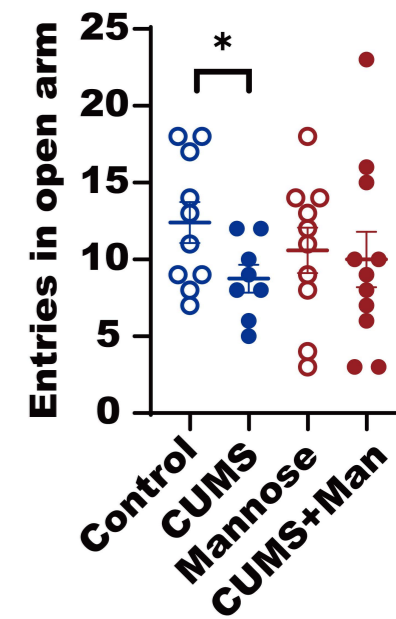

Supplement: Supplementary file 5 — Figure S5 [file 41398_2023_2636_MOESM5_ESM.pdf]

**A**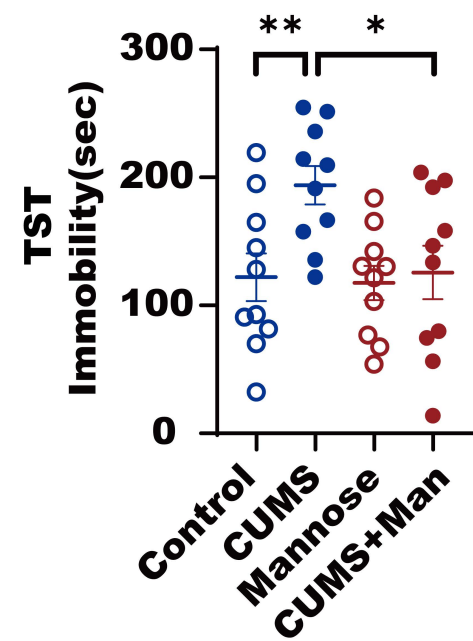**B**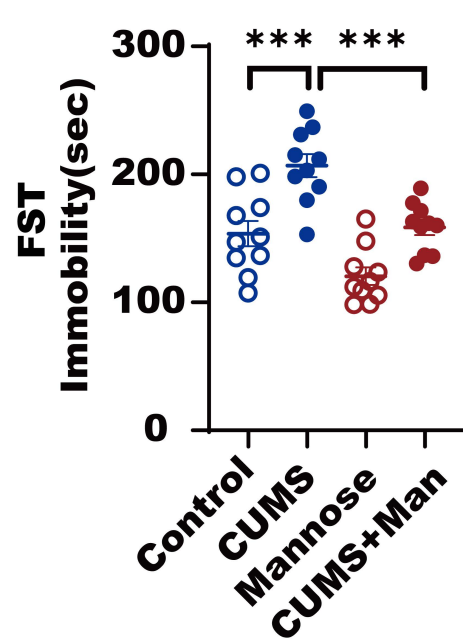**C**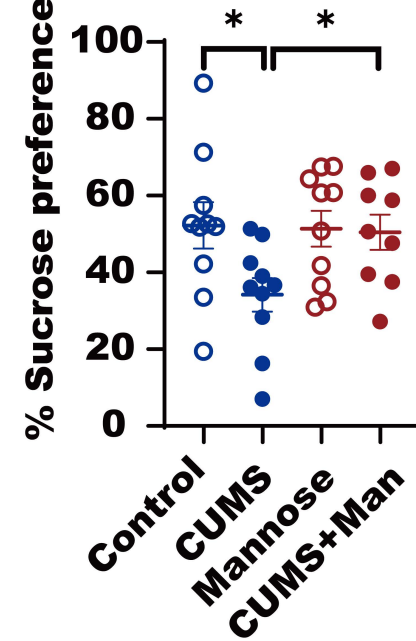**D**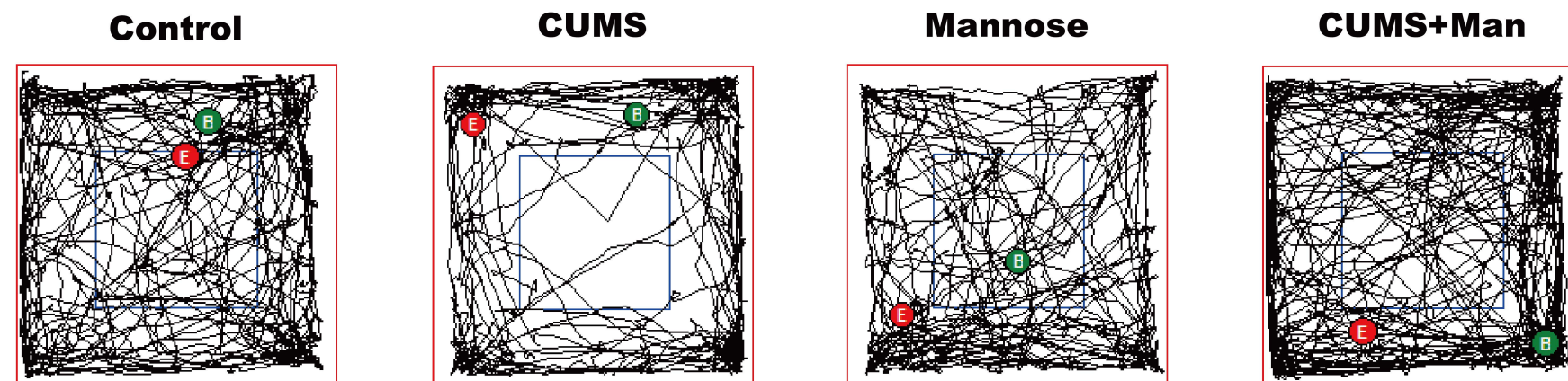**E**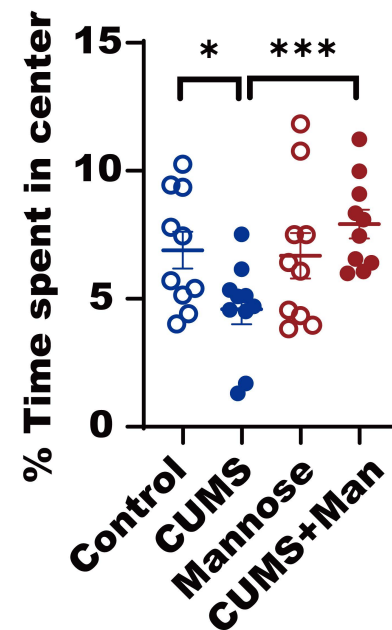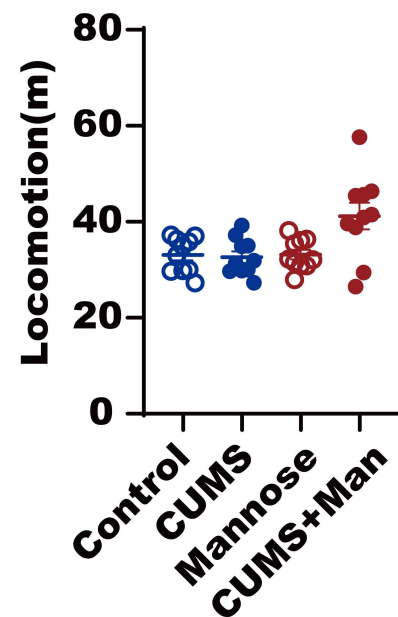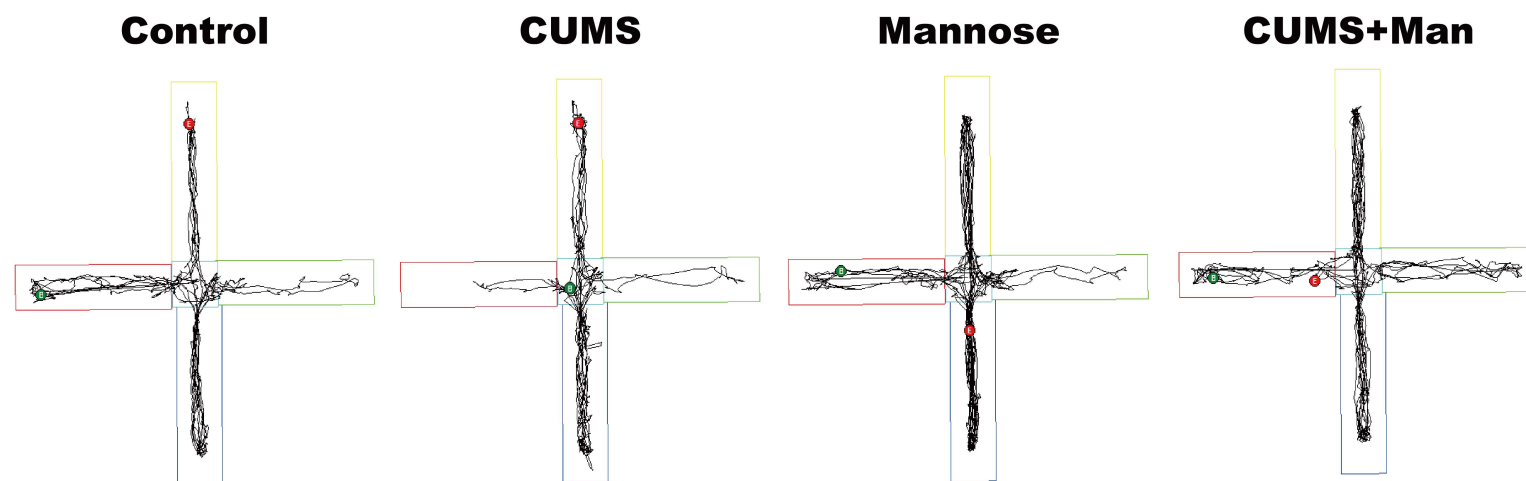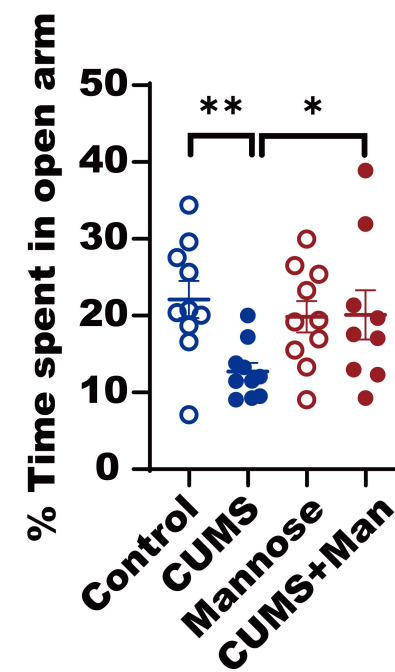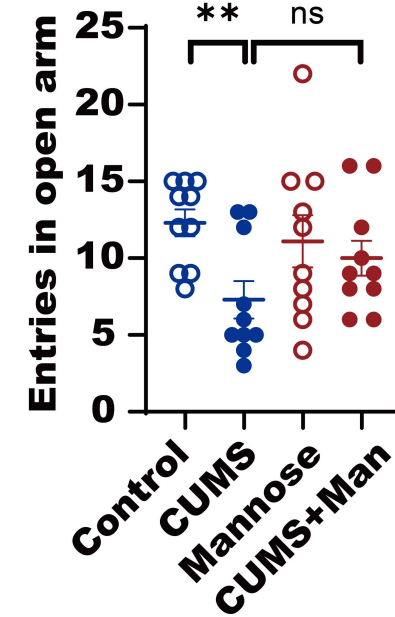

Supplement: Supplementary file 6 — Figure S6 [file 41398_2023_2636_MOESM6_ESM.pdf]

**A**

♀

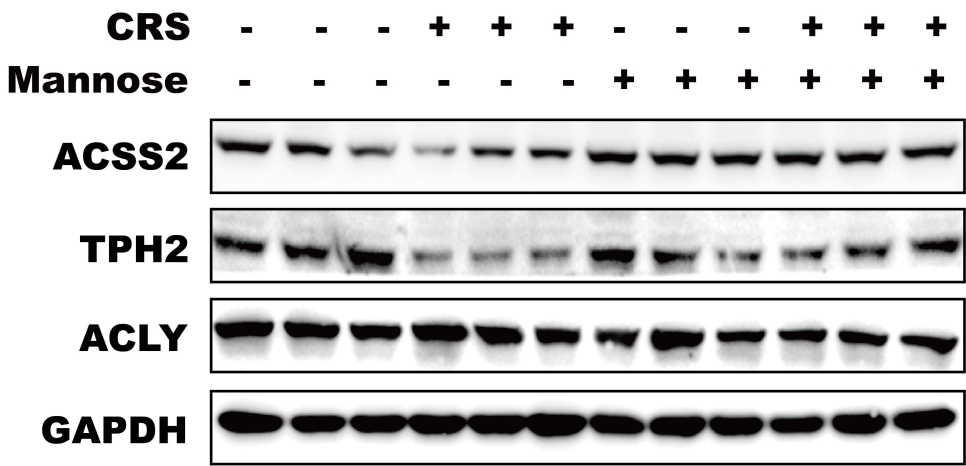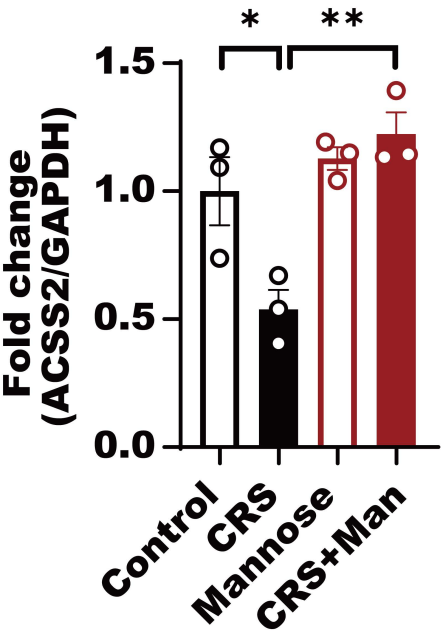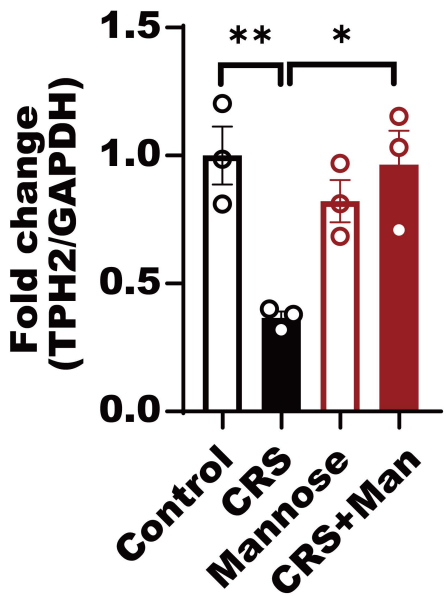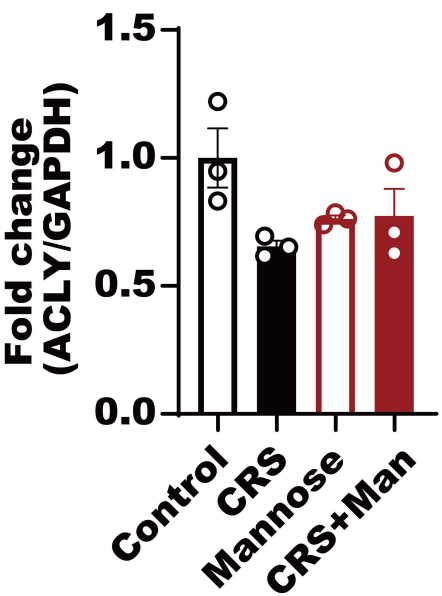

**B**

♂

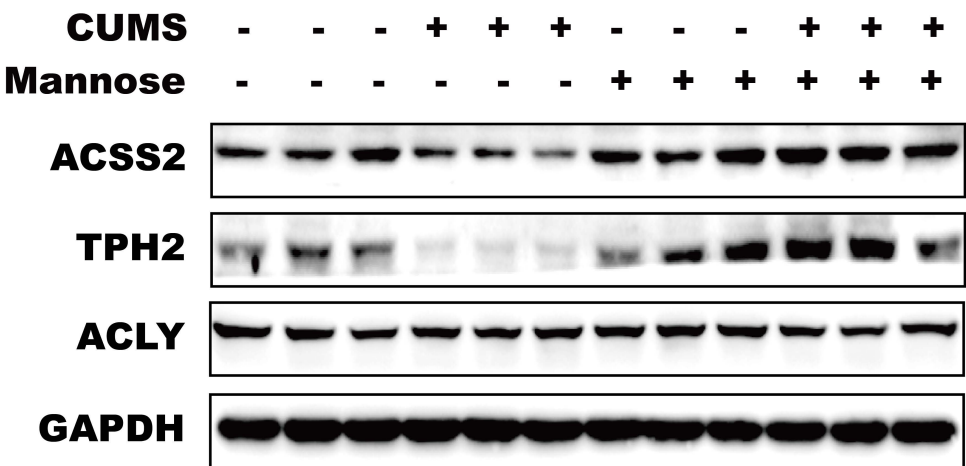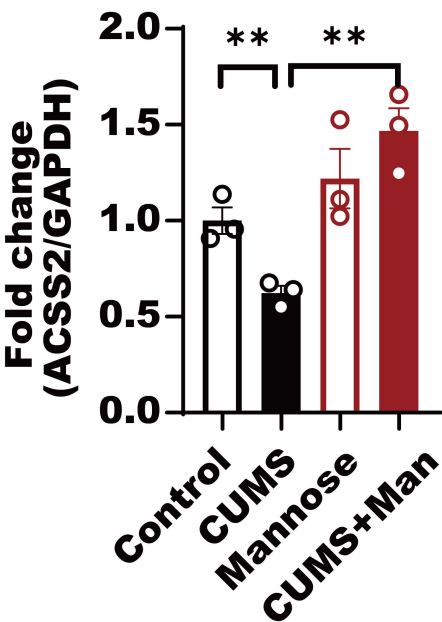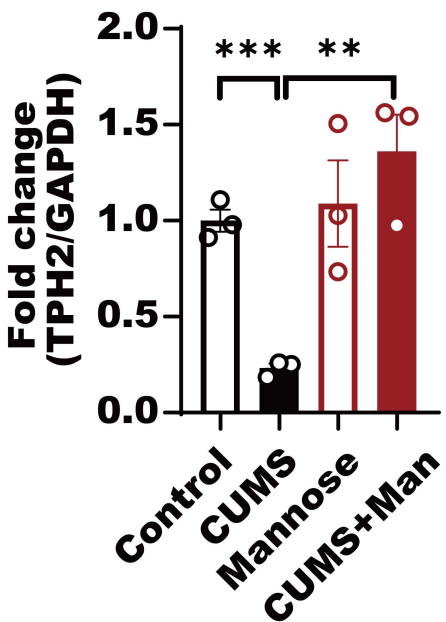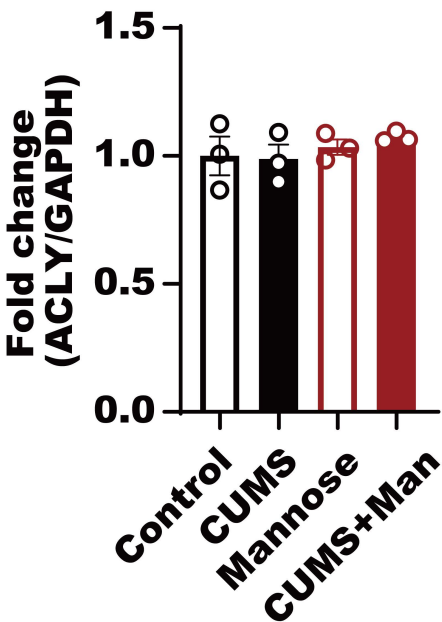

**C**

♀

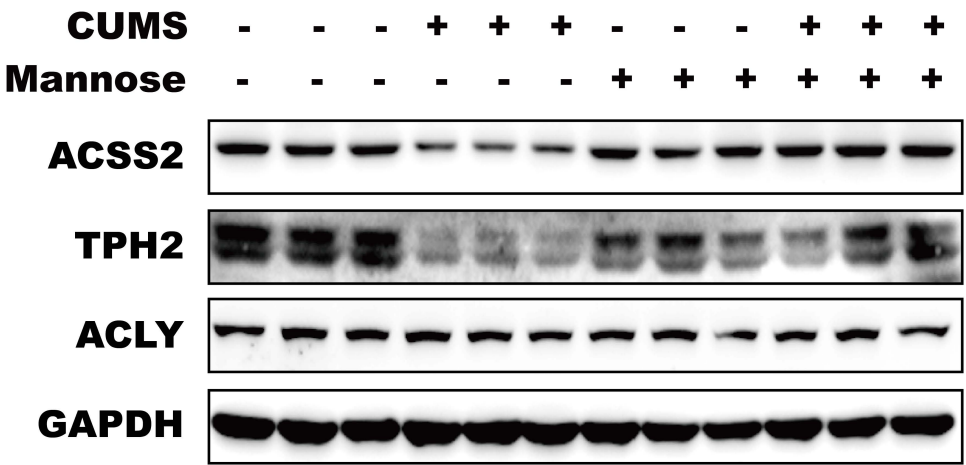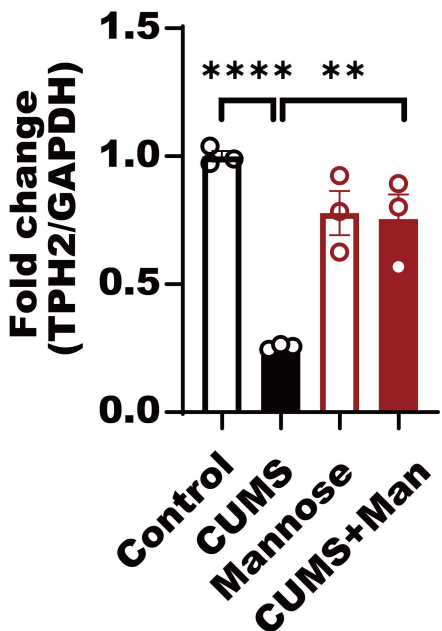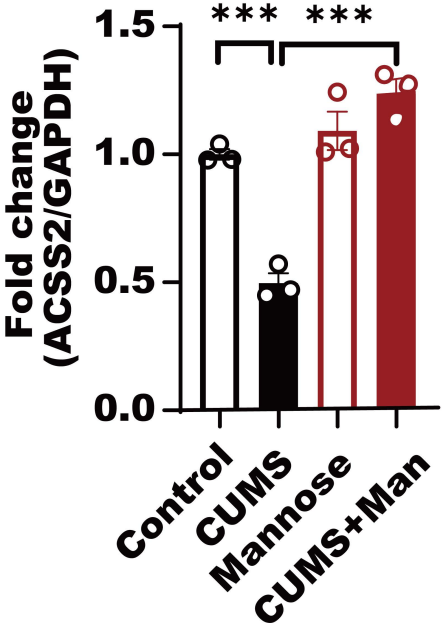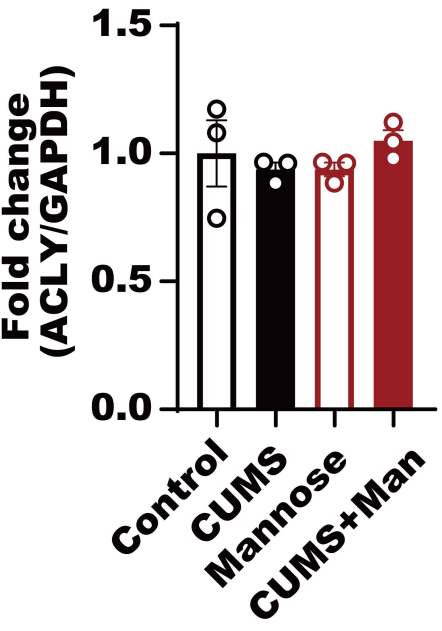

Supplement: Supplementary file 7 — Figure S7 [file 41398_2023_2636_MOESM7_ESM.pdf]

**A**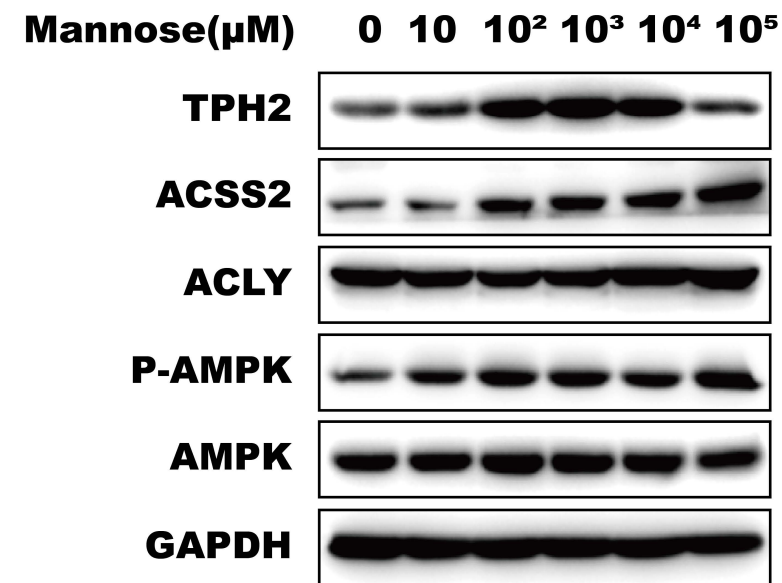**B**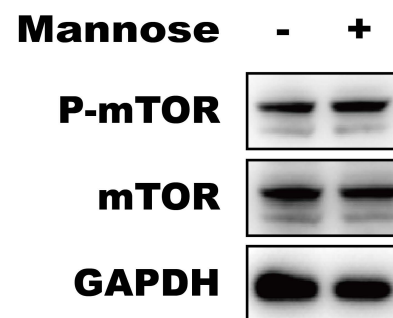**C**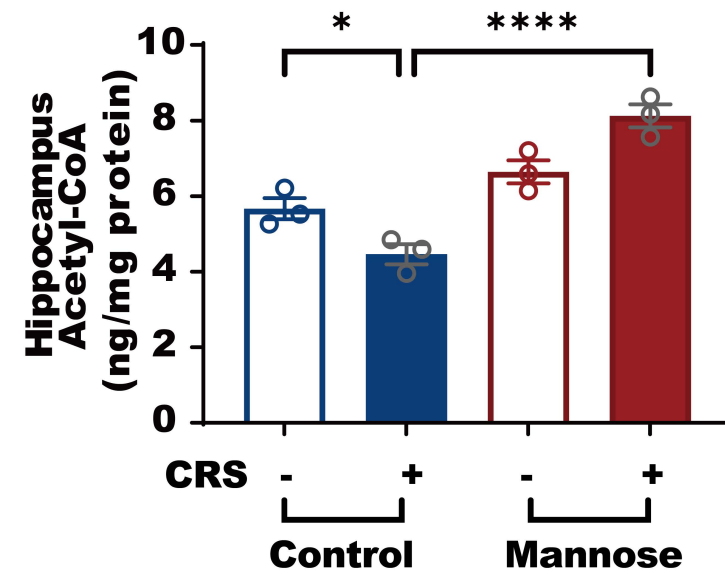**D**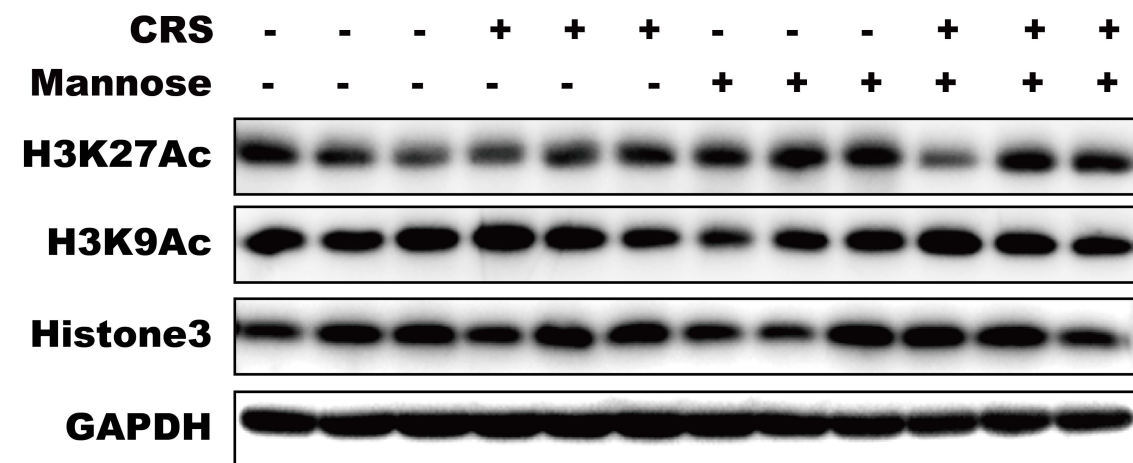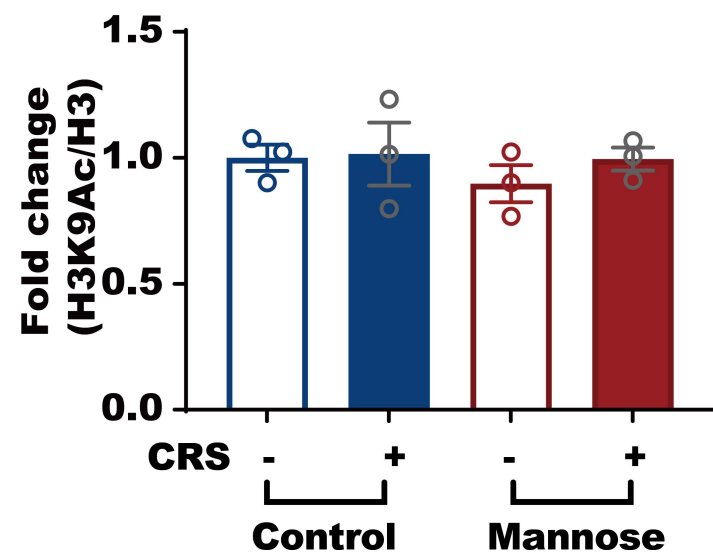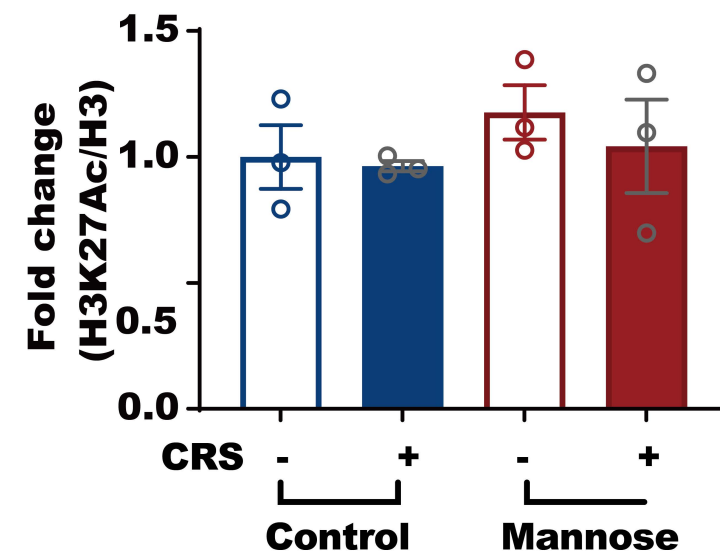**E**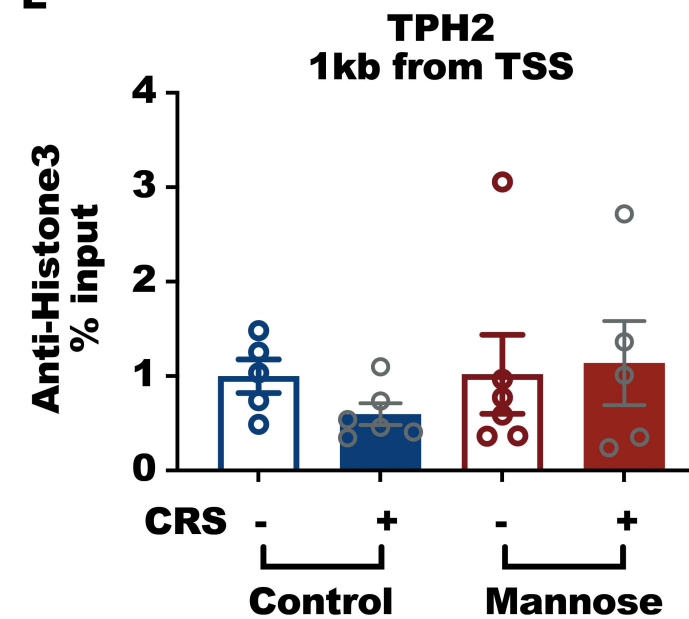

Supplement: Supplementary file 8 — Figure S8 [file 41398_2023_2636_MOESM8_ESM.pdf]
