# Supplementary material for: D-mannose is a rapid inducer of ACSS2 to trigger rapid and long-lasting antidepressant responses through augmenting BDNF and TPH2 levels
Source: Transl Psychiatry. 2023 Nov 1;13:338. doi: 10.1038/s41398-023-02636-7 (PMC10620401; doi:10.1038/s41398-023-02636-7)
Supplement: Supplementary file 9 — Figure S9 [file 41398_2023_2636_MOESM9_ESM.pdf]

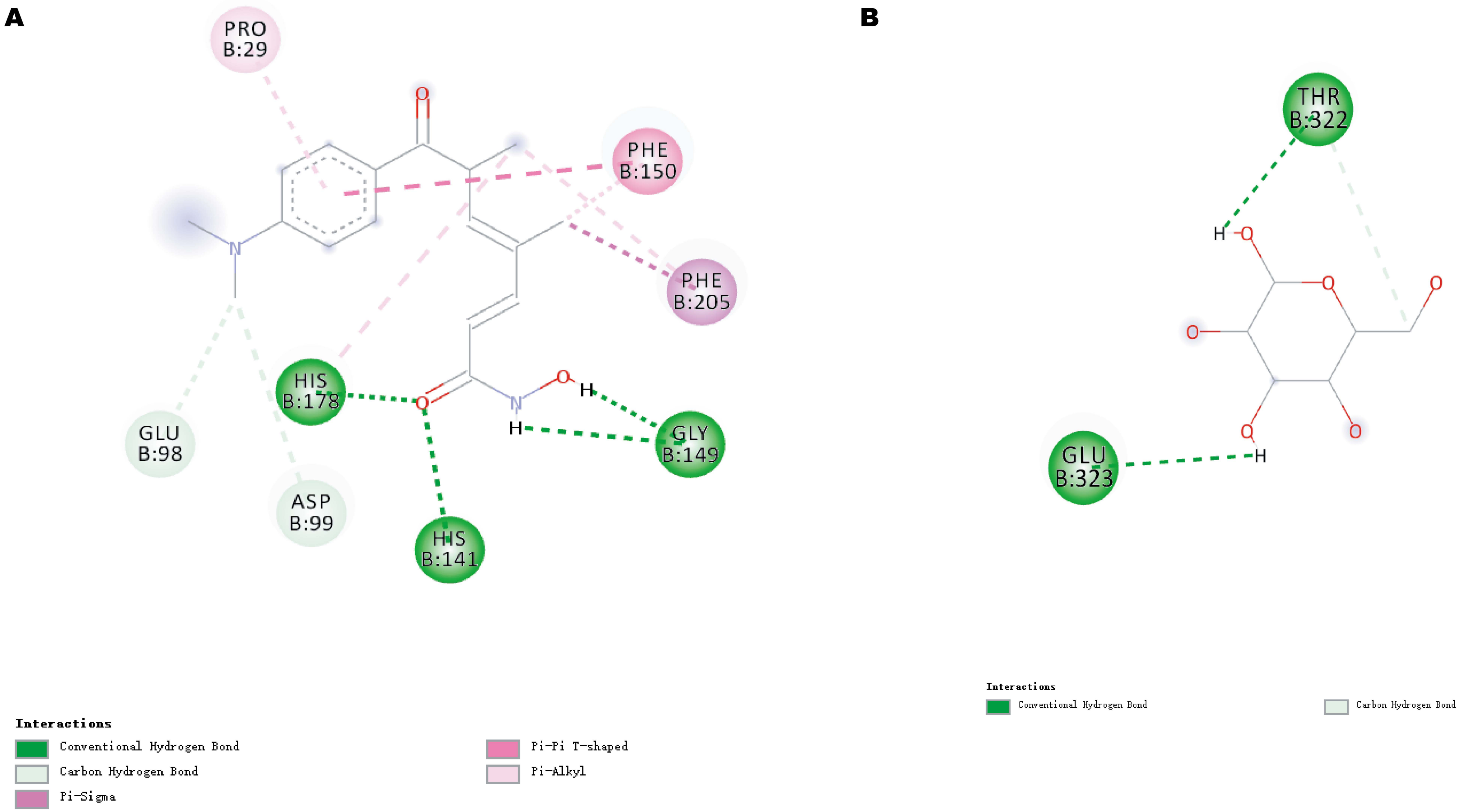

TSA and HDAC1

Mannose and HDAC1
