# Supplementary material for: D-mannose is a rapid inducer of ACSS2 to trigger rapid and long-lasting antidepressant responses through augmenting BDNF and TPH2 levels
Source: Transl Psychiatry. 2023 Nov 1;13:338. doi: 10.1038/s41398-023-02636-7 (PMC10620401; doi:10.1038/s41398-023-02636-7)
Supplement: Supplementary file 10 — supplementary figure legends [file 41398_2023_2636_MOESM10_ESM.docx]

Figure S1. **Lack of ACSS2 causes defects of synaptic formation and reduced levels of BDNF and TPH2 to induce depressive symptoms**

(A) The body weight of 8-week-old wild type, *Acss2^-/-^* and *Acss2^+/-^* male mice(n=8) was calculated.

(B) The food intake of 8-week-old wild type, *Acss2^-/-^* and *Acss2^+/-^* male mice(n=8) was calculated.

(C) The water intake of 8-week-old wild type, *Acss2^-/-^* and *Acss2^+/-^* male mice(n=8) was calculated.

(D) Raw traces of male mice in the OFT were shown. Total distance traveled in the OFT and time spent exploring the center area in the open field test from male mice in individual animals from wild type, Acss2-/- and Acss2+/- groups (n≥8).

(E) Raw traces of male mice in the EPM were shown. Time spent in the open arms and probability of entering open arms in the Elevated Plus Maze test from male mice in individual animals from wild type, Acss2-/- and Acss2+/- groups (n≥8).

(F) Raw traces of male mice in the OFT were shown. Total distance traveled in the OFT and time spent exploring the center area in the open field test from male mice in individual animals from si-NC and si-ACSS2 groups (n≥8).

(G) Raw traces of male mice in the EPM were shown. Time spent in the open arms and probability of entering open arms in the Elevated Plus Maze test from male mice in individual animals from si-NC and si-ACSS2 groups (n≥8).

(H) C57BL/6J male mice suffered from chronic binding stress for 4 weeks, and TST behaviour test was detected on Day29.

Figure S2. **Acute D-mannose administration possesses fast-acting antidepressant activity via ACSS2**

(A) 125ul 2mM CY5-Glucose was injected into male mice via tail vein, the intensity and distribution of red fluorescence were detected by the small animal three-dimensional live imaging system at 5min, 1h, 3h, 12 h and 24h after injection.

(B) Left half of hippocampus weights from Control, D-Mannose groups of male mice were analyzed (n=7).

(C) The enzymatic activities of ALT and AST in serum of WT male mice with or without 10% D-Mannose administration were determined. The levels of ALP, LDH, TP, ALB and GloB in serum of these male mice were also measured (n=8).

(D) The levels of Glucose, Urea, Cr and UA in serum of these male mice were measured (n=8).

(E) Representative images of H&E staining of liver in male mice from Vehicle and D-Mannose groups.

(F) Analysis of Glutamate content of HIP by ELISA in WT male mice with or without 10% D-Mannose (n=9).

(G) Representative photomicrographs of dendritic spines from DG granular cells, scale bar, 10 μm. And Spine density in dendrites of DG granular cells, (Vehicle n = 3 and D-Mannose n = 3 per group). Values are indicated as mean ± SD one-way ANOVA and multiple comparison test, *P ≤ 0.05 **P ≤ 0.01. All the experiments were repeated at least three times independently.

Figure S3. **Oral administration of D-mannose has long-lasting antidepressant actions by activating the ACSS2-TPH2 axis in mice with CRS-induced depressive-like behaviors**

(A) ATP and Reactive Oxygen species (ROS) examination (B) in individual animals from Control, CRS-Control, D-Mannose, CRS-D-Mannose groups (n=4-5). The ATP and ROS levels were normalized to those in Control mice.

(C) Left half of hippocampus weights from Control, CRS-Control, D-Mannose, CRS-D-Mannose groups of male mice were analyzed (n=9-10).

(D) The enzymatic activities of ALT and AST in serum of WT male mice with or without 10% D-Mannose administration were determined. The levels of ALP, LDH, TP, ALB and GloB in serum of these male mice were also measured (n≥8).

(E) The levels of Glucose, Urea, Cr and UA in serum of male mice were measured

(n≥8).

(F) Representative images of H&E staining of liver in male mice from Vehicle and D-Mannose groups.

(G) The enzymatic activities of ALT and AST in serum of WT female mice with or without 10% D-Mannose administration were determined. The levels of ALP, LDH, TP, ALB and GloB in serum of female mice were also measured (n≥8).

(H) The levels of Glucose, Urea, Cr and UA in serum of these mice were measured

(n≥8).

(I) Representative images of H&E staining of liver in female mice from Vehicle and D-Mannose groups.

Figure S4. **Oral administration of D-Mannose can reverse anxiety/depressive-like behaviour in female mice with CRS-induced depressive-like behaviors.**

(A) Immobility time in the tail suspension test from female mice in individual animals from Control, CRS and CRS-D-Mannose groups (n≥8).

(B) Immobility time in the forced swimming test from female mice in individual animals from Control, CRS and CRS-D-Mannose groups (n≥8).

(C) Sucrose consumption in the sucrose preference test from female mice in individual animals from Control, CRS and CRS-D-Mannose groups (n≥8).

(D) Raw traces of female mice in the OFT were shown. Total distance traveled in the OFT and time spent exploring the center area in the open field test from female mice in individual animals from Control, CRS and CRS-D-Mannose groups (n≥8).

(E) Raw traces of female mice in the EPM were shown. Time spent in the open arms and probability of entering open arms in the Elevated Plus Maze test from female mice in individual animals from Control, CRS and CRS-D-Mannose groups (n≥8).

Figure S5. **Oral administration of D-Mannose can reverse anxiety/depressive-like behaviour in male mice with CUMS-induced depressive-like behaviors.**

(A) Immobility time in the tail suspension test from male mice in individual animals from Control, CUMS and CUMS-D-Mannose groups (n≥8).

(B) Immobility time in the forced swimming test from male mice in individual animals from Control, CUMS and CUMS-D-Mannose groups (n≥8).

(C) Sucrose consumption in the sucrose preference test from male mice in individual animals from Control, CUMS and CRS-D-Mannose groups (n≥8).

(D) Raw traces of male mice in the OFT were shown. Total distance traveled in the OFT and time spent exploring the center area in the open field test from male mice in individual animals from Control, CUMS and CUMS-D-Mannose groups (n≥8).

(E) Raw traces of male mice in the EPM were shown. Time spent in the open arms and probability of entering open arms in the Elevated Plus Maze test from male mice in individual animals from Control, CUMS and CUMS-D-Mannose groups (n≥8).

Figure S6. **Oral administration of D-Mannose can reverse depressive/anxiety-like behaviour in female mice with CUMS-induced depressive-like behaviors.**

(A) Immobility time in the tail suspension test from female mice in individual animals from Control, CUMS and CUMS-D-Mannose groups (n≥8).

(B) Immobility time in the forced swimming test from female mice in individual animals from Control, CUMS and CUMS-D-Mannose groups (n≥8).

(C) Sucrose consumption in the sucrose preference test from female mice in individual animals from Control, CUMS and CRS-D-Mannose groups (n≥8).

(D) Raw traces of female mice in the OFT were shown. Total distance traveled in the OFT and time spent exploring the center area in the open field test from female mice in individual animals from Control, CUMS and CUMS-D-Mannose groups (n≥8).

(E) Raw traces of female mice in the EPM were shown. Time spent in the open arms and probability of entering open arms in the Elevated Plus Maze test from female mice in individual animals from Control, CUMS and CUMS-D-Mannose groups (n≥8).

Figure S7. **Oral administration of D-Mannose has long-lasting antidepressant actions via activating ACSS2-TPH2 axis in mice with depressive-like behaviors.**

(A) Representative immunoblots and quantification of hippocampal ACSS2, TPH2, ACLY protein levels normalized to loading controls (n=3) from female mice exposure to CRS and D-Mannose.

(B) Representative immunoblots and quantification of hippocampal ACSS2, TPH2, ACLY protein levels normalized to loading controls (n=3) from male mice exposure to CUMS and D-Mannose.

(C) Representative immunoblots and quantification of hippocampal ACSS2, TPH2, ACLY protein levels normalized to loading controls (n=3) from female mice exposure to CUMS and D-Mannose.

Figure S8. **ACSS2 promotes *TPH2* histone acetylation and transcription in response to D-Mannose-mediated AMPK activation**

(A) SH-SY5Y cells were incubated by increasing D-Mannose (0-100 mM) for 24 hours. Immunoblotting assays were conducted with the indicated antibodies.

(B) Immunoblotting analyses of SH-SY5Y cells with or without 1 mM D-Mannose for 24 hours were performed with anti-p-mTOR and anti-mTOR antibodies.

(C) Analysis of Acetyl-CoA levels in Hip of male mice (n=15, five mice per group were combined as one sample for Acetyl-CoA evaluation) in presence of CRS and D-Mannose.

(D) Representative immunoblots of hippocampal Histone H3K27AC, histone H3K9AC and histone H3 protein levels normalized to loading controls (n=3) from male mice exposure to CRS and D-Mannose.

(E) The Hip tissues from Control, CRS-Control, D-Mannose and CRS-D-Mannose groups were collected and tissues of two male mice per group were added together to be a sample for chromatin immunoprecipitation (CHIP) assay. CHIP analyses using an anti-Histone H3 antibody were performed. The histogram shows the amount of immunoprecipitated DNA expressed as a percentage of the total input DNA. The data are presented as the mean ± SEM of six samples of male mice (n=5-6).

Figure S9. **Molecular docking of D-Mannose with HDAC1**

(A) Molecular docking of TSA (Generated by ChemDraw Professional software) and HDAC1(5ICN. Protein Data Bank (PDB)) by Auto-dock server: The image shows the amino acid in the interaction face between TSA and HDAC1. Phe150, Phe205, Gly149, His141, His178, Asp99, Glu98 and Pro29 of HDAC1 participate in binding TSA.

(B) Molecular docking of D-Mannose (Generated by ChemDraw Professional software) and HDAC1(5ICN. Protein Data Bank (PDB)) by Auto-dock server: The image shows the amino acid in the interaction face between D-Mannose and HDAC1. Thr322 and Glu323 of HDAC1 participate in binding D-Mannose.
