# Supplementary material for: D-mannose is a rapid inducer of ACSS2 to trigger rapid and long-lasting antidepressant responses through augmenting BDNF and TPH2 levels
Source: Transl Psychiatry. 2023 Nov 1;13:338. doi: 10.1038/s41398-023-02636-7 (PMC10620401; doi:10.1038/s41398-023-02636-7)
Supplement: Supplementary file 11 — TableS1-KEY RESOURCES TABLE [file 41398_2023_2636_MOESM11_ESM.docx]

**MATERIALS AND METHODS**

**KEY RESOURCES TABLE**

| **REAGENT or RESOURCE** | **SOURCE** | **IDENTIFIER** |
| --- | --- | --- |
| **Antibodies** |  |  |
| Anti-ACSS2 | Santa Cruz | 398559 |
| Anti-BDNF | Abcam | ab108319 |
| Anti-TPH2 | Abcam | Ab111828 |
| Anti-ACLY | Santa Cruz | sc-517267 |
| Anti-AMPKα | CST | 5831 |
| Anti-AMPKα-p-T172 | CST | 2535S |
| Anti-mTOR | CST | 2983S |
| Anti-P-mTOR | CST | 5536S |
| Anti-P-EF2 | Abcam | ab82981 |
| Anti-EF2 | Santa Cruz | sc-166415 |
| Anti-Synapsin Ia/b | Santa Cruz | sc-376623 |
| Anti-PSD95 | Santa Cruz | sc-32290 |
| Anti-EAAT2 | Santa Cruz | sc-365634 |
| Anti-ACC | CST | 3676S |
| Anti-P-ACC | CST | 11818S |
| Anti-LAMP1 | CST | 9091S |
| Anti-AXIN | CST | 2074S |
| Anti-CAMKK2 | CST | 16810S |
| Anti-Histone H3 | CST | 4620S |
| Anti-Histone H3K9AC | CST | 9649S |
| Anti-Histone H3K27AC | CST | 4353S |
| Anti-GAPDH | ZSGB-BIO | TA-08 |
| Anti-β-Actin | ZSGB-BIO | TA-09 |
| Anti-Lamin-B | Proteintech | 66095-1-Ig |
| Alexa Fluor® 594 | Abcam | ab150080 |
| HPLC and GC standards |  |  |
| Serotonin | Sigma-Aldrich | Cat#H9523 |
| L-Tryptophan | Yuanye Bio | Cat#B23263 |
| 5-HTP | Yuanye Bio | Cat#B24406 |
| 5-HIAA | Yuanye Bio | Cat#B27047 |
| Chemicals and inhibitors |  |  |
| PCPA | Sigma-Aldrich | C3635 |
| FITC-D-Mannose | QiyueBio | QY-C-FDG5 |
| CY5-D-Glucose | QiyueBio | QY-C-CDP41 |
| D-Mannose | Macklin | D-813082 |
| D-Glucose | HUSHI | 10010518 |
| D-Fructose | Macklin | D809612 |
| Protease inhibitor cocktail | Bimake | B14002 |
| PVDF membranes | Millipore | IPVH00010 |
| Protein A/G-Sepharose | Santa Cruz | sc-2003 |
| Lipofectamine 2000 | Invitrogen | Cat#11668019 |
| ACSS2 inhibitor | Selleck | S8588 |
| AICAR | Selleck | S1802 |
| Compound C | Selleck | S7306 |
| STO-609 | Selleck | S8274 |
| Fluo-4, AM | Thermo Fisher | F14201 |
| CoA | Sigma-Aldrich | Cat#C4282 |
| DAPI | Invitrogen | Cat#S36964 |
| Lysosensor | Invitrogen | Cat#L7535 |
| Digitonin | Sigma-Aldrich | Cat#D141 |
| Critical Commercial Assays |  |  |
| Mouse 5-HT ELISA assay kit | Jianglai Bio | Cat#JL12087 |
| Mouse 5-HT ELISA assay kit | Fine Test | Cat#EM1465 |
| Mouse DA ELISA assay kit | Fine Test | Cat# EM1712 |
| Mouse NE ELISA assay kit | Fine Test | Cat# EU2565 |
| Mouse BDNF ELISA assay kit | Fine Test | Cat# EM0020 |
| Human acetyl-CoA ELISA assay kit | Jianglai Bio | Cat#JL32777 |
| ATP Assay Kit | Beyotime | Cat#S0026 |
| Lysosome isolation Kit | Bestbio | Cat #BB31452 |
| Endo-free plasmid Midi-Kit | CWBio | Cat#CW2105S |
| eECL Western Blot Kit | Millipore | Cat#69078 |
| SimpleChIP® Plus Sonication Chromatin IP Kit | CST | Cat#56383 |
| The Nuclear/Cytosol Fractionation Kit | Thermo | Cat#78833 |
| Dual luciferase assay system | Vazyme | Cat#DL101-01 |
| Experimental Models: Cell Lines |  |  |
| Cell line: HEK293T | Shanghai Cell Bank of Chinese Aca- demy of Sciences | GNHu17 |
| Cell line: SH-SY5Y | ATCC | CRL-2266 |
| Cell line: Hela | ATCC | CRM-CCL-2 |
| Experimental Models: Organism |  |  |
| Mouse: C57BL/6J | Charles River | N/A |
| Bacteria: *Escherichia coli* DH5α | Zhuangmeng Bio | ZK206 |
| Oligonucleotides |  | |
| Primers |  | |
| Primers used for quantitative PCR, see Table S2 | This study | N/A |
| Primers used for CHIP assay, see Table S2 | This study | N/A |
| Primers used for plasmids construction, see Table S2 | This study | N/A |
| Recombinant DNA |  |  |
| pGL3 basic | Promega | Cat#E1751 |
| PPRE X3-TK-luc | addgene | 1015 |
| PGL3-TPH2 promoter-Luciferase (Human) | This study | N/A |
| PGL3-TPH2 promoter-Luciferase (Mouse) | This study | N/A |
| PCMV-ACSS2 | This study | N/A |
| PGL3-TPH2 | This study | N/A |
| Software |  |  |
| Image J |  | https://imagej.en.softonic.com |
| Zeiss Zen | Zeiss | https://www.zeiss.com |
| GraphPad Prism | GraphPad | https://www.graphpad.com/ |
| iMARAS | iMARAS |  |
| FlowJo | FlowJo |  |
| Other |  |  |
| Develosil ODS-UG-3 column (7.5 cm×4.6 mm i.d.) | Nomura Chemical | Japan |
| Kinetex 2.6μ C18 100×4.6 mm column | Phenomenex | UK |
